# EV71 infection alters the lipid composition of human rhabdomyosarcoma (RD) cells-derived extracellular vesicles

**DOI:** 10.3389/fmicb.2024.1430052

**Published:** 2024-09-05

**Authors:** Lingxiang Mao, Qing Gao, Yuxuan Shen, Chenxuan Bao, Huayuan Xiang, Qiaoqiao Chen, Qianqian Gao, Feng Huang, Wenyuan He, Jianjun Wang

**Affiliations:** ^1^Department of Laboratory Medicine, Affiliated Kunshan Hospital of Jiangsu University, Kunshan, Jiangsu, China; ^2^Medical College, Yangzhou University, Yangzhou, Jiangsu, China; ^3^Department of Laboratory Medicine, Affiliated People’s Hospital of Jiangsu University, Zhenjiang, Jiangsu, China

**Keywords:** lipidomics, lipid, extracellular vesicles, EV71, SEC

## Abstract

Previous studies demonstrated that EV71-infected cells secrete extracellular vesicles (EVs), facilitating the transfer of viral components to recipient cells and thereby promoting virus spread. Considering lipid signaling plays a crucial role in EVs-mediated cell-to-cell communication, we compared the lipid profile of EVs secreted from uninfected and EV71-infected cells (EVs-Mock and EVs-EV71) using the human rhabdomyosarcoma (RD) cell model. These two groups of EVs were purified by using size exclusion chromatography (SEC), respectively, and evaluated by transmission electron microscopy (TEM), nanoparticle tracking technology (NTA), and Western blotting (WB). In-depth lipidomic analysis of EVs identified 1705 lipid molecules belonging to 43 lipid classes. The data showed a significant increase in the lipid content of EVs after EV71 infection. Meanwhile, we deeply analyzed the changes in lipids and screened for lipid molecules with significant differences compared EVs-EV71 with EVs-Mock EVs. Altogether, we report the alterations in the lipid profile of EVs derived from RD-cells after EV71 infection, which may affect the function of the EVs in the recipient cells.

## 1 Introduction

Extracellular vesicles (EVs) refer to particles that are released from cells, are delimited by a lipid bilayer, and cannot replicate on their own (i.e., do not contain a functional nucleus) (Welsh et al., 2024). All cellular organisms, prokaryotes, and eukaryotes, release EVs (Choi et al., 2015). Depending on their biogenesis pathways, two main types of small EVs can be distinguished, ectosomes and exosomes. Ectosomes are vesicles generated by the direct outward budding of the plasma membrane, which produces microvesicles, microparticles, and large vesicles ranging in size from 50 nm to 1 μm in diameter (Cocucci and Meldolesi, 2015). In contrast, exosomes, which are EVs with diameters ranging from about 30 to 160 nm (average about 100 nm), are small EVs of endosomal origin. The main pathway of exosome secretion is the formation of multivesicular bodies (MVBs) by successive invaginations of the plasma membrane, followed by fusion of the MVBs with the cytoplasmic membrane and release of intraluminal vesicles (ILVs) within the MVBs into the extracellular space to form what we call exosomes (van Niel et al., 2018; Kalluri and LeBleu, 2020). It is the complex process of material exchange between MVBs and other intracellular organelles during the biogenesis of exosomes that allows exosomes to carry an abundance of cargo (Wortzel et al., 2019). Although various types of extracellular vesicles are theoretically distinct from each other, there is no technique to completely separate these biogenesis-based EV subtypes currently (van Niel et al., 2018). Therefore, the International Society for Extracellular Vesicles on the Minimal Information for Studies of Extracellular Vesicles 2018 (MISEV 2018) suggests the use of alternative terms such as “small EVs” (< 200 nm) or “large EVs” (> 200 nm) (Théry et al., 2018).

As an essential component of intercellular communication, EVs carry out biological tasks by communicating data at the level of the cargo they carry, which may include proteins (such as cytokines, membrane receptors, and receptor ligands), lipids, and nucleic acids (such as DNA, mRNA, lncRNA, and microRNA) (Yoon et al., 2014). By transferring functional cargo to receptor cells, EVs can control the physiological and pathological states of cells, as well as contribute to the onset and progression of a wide range of disorders (de Toledo Martins et al., 2019). Studies have shown that many viruses employ a dissemination strategy mediated by the EVs (Meckes et al., 2010; Pegtel et al., 2010). Viruses can release and disseminate their complete or partial nucleic acids, proteins, receptor cell-regulating small molecules (mRNA, miRNA, lncRNA, cirRNA, etc.), and even intact viral particles from host cells to uninfected recipient cells by EVs (Anderson et al., 2016). The viruses disguise themselves within EVs, much like a Trojan horse, secreting themselves from the donor cells into the extracellular environment (Narayanan et al., 2013), just like the human immunodeficiency virus (HIV) (Nwankwo and Seker, 2013), ZIKA virus (Joob and Wiwanitkit, 2019), Ebola Virus (Altan-Bonnet et al., 2019), and so on.

Enterovirus 71 (EV71) is a positive-stranded RNA virus from the enterovirus genus of the *Picornaviridae* family (Solomon et al., 2010). The EV71 particle is non-enveloped, and symmetrical, with a 20–30 nm icosahedral capsid. As a non-enveloped virus, its genomic RNA is encapsidated in a capsid, which consists of four structural proteins, VP1, VP2, VP3, and VP4, with VP1-VP3 being exposed on the outside of the capsid and VP4 is located on the inside of the capsid (Wang and Li, 2019). As with many other non-enveloped viruses, EV71 has to induce lysis of host cells to release viral particles to spread infection. However, previous studies have shown that EVs can act as vehicles for the intercellular spread of EV71 by a non-lytic strategy, enabling the virus to evade immune surveillance and further propagate in uninfected cells (Gu et al., 2020a).

It is worth noting that lipids are one of the most abundant components of EVs, serving a crucial function in structural (membrane stability and rigidity), formation, and release processes, as well as being effective regulators of cellular signaling (Choi et al., 2013; Llorente et al., 2013; Pienimaeki-Roemer et al., 2015). However, current studies have focused mainly on the protein and nucleic acid of EVs, and the research on the lipids of EVs is still in its infancy. In order to study the production and mechanism of action of these vesicles, it is necessary to understand the lipid composition in EVs better. Although ultracentrifugation was the most used technique for EV isolation, it is difficult to separate free EV71 virion from the EVs derived from the EV71-infected cell (EVs-EV71) because of their close density. After differential velocity centrifugation, we further purified the EVs using a size-based separation method-size exclusion chromatography (SEC). We chose the SEC column (qEV/35 nm) packed with a porous, polysaccharide resin with a pore size < 35 nm to separate EVs-EV71 (> 35 nm) from the EV71 virion (20–30 nm) completely. After the separation, we used the lipidomic research methods to compare EVs’ lipid profiles derived from uninfected and EV71-infected cells, respectively.

## 2 Materials and methods

### 2.1 Cells and viruses

RD cells were grown in Dulbecco modified Eagle medium (DMEM), supplemented with 10% fetal bovine serum (FBS), 100 μg/mL penicillin, and 100 μg/mL streptomycin at 37 lbecco modi_2_ incubator. The cell line was obtained from ATCC.

### 2.2 Virus infection

Enteroviruses EV71 (GenBank accession number OP91657) were preserved in our lab. The virus was amplified using RD cells and was harvested by three freeze-thaw cycles and low-speed centrifugation at 100*g* for 30 min. Infectious virus titers were determined by TCID50 assay. Cells were infected with EV71 at a multiplicity of infection (MOI) of 1 and incubated for 1 h at 37 °C and 5% CO2.

### 2.3 EVs purification

EVs were prepared from RD cell cultures (initial seeding: 410^6^ cells/10 cm dish). After 70–80% confluence of RD cells, the normal cell growth medium was replaced with an exosome-depleted cell growth medium consisting of DMEM, 10% exosome-depleted FBS (Gibco), and 1% penicillin/streptomycin, and collected after one day of cell culture. The collected culture solution was first spun at 300g for 10 min at room temperature to remove suspended cells, then spun at 2,000g for 10 min at 4 °C to remove cell debris, followed by subsequent purification steps. The supernatant was passed through a 0.22 μm filter (Merck Millipore, MA, USA) and then transferred to Amicon^®^ Ultra-100 centrifugal filter devices (Merck Millipore, MA, USA) and centrifuged at 1,000*g* for 30 min at 4 °C to obtain a concentrate. Finally, a qEV original 35 nm column (Izon Science, MA, USA) was used according to the manufacturer’s instructions.^1^ As the sample passes through the column under gravity, smaller particles enter the resin pores on their way down and their exit from the column is delayed. According to the size exclusion principle, the first to flow out of the qEV/35 nm column is the large-particle fraction (> 300 nm), while small and medium particles flow out slowly. The molecules smaller than 35 nm enter the pores of the resin and are delayed in their passage through the column, eluting mainly in later fractions. We collected the EVs-enriched medium fraction (35–300 nm) and discarded later fraction containing the EV71 virions (< 35 nm).

### 2.4 Transmission electron microscopy (TEM)

Samples (20 μL) were adsorbed onto the surface of a glow-discharged, 400-mesh carbon-coated copper grid for 1 min and subsequently stained with a 3% phosphotungstic acid negative staining solution for 5 min. The copper mesh was then dried under an incandescent lamp and imaged using TEM.

### 2.5 Nanoparticle tracking analysis (NTA)

Samples were diluted appropriately and the concentration and size distributions were measured using a NanoSightLM10 (Malvern Instruments Ltd, Worcestershire, UK), and the data were statistically analyzed by the software to determine the size dependence of the nanoparticles.

### 2.6 Western blot (WB)

EVs were collected and lysed with radioimmunoprecipitation assay (RIPA) buffer (Kangwei Century), and extracted proteins were separated by sodium dodecyl sulfate-polyacrylamide gel electrophoresis (SDS-PAGE) and transferred to a polyvinylidene fluoride (PVDF) membrane. The membranes were then blocked in containing 5% non-fat dry milk and 0.1% Tween-20 and incubated with primary antibody overnight at 4 °C. The primary antibodies were used: anti-Calnexin, anti-HSP70, anti-TSG101, and anti-CD63 antibodies. After washing three times per 10 min, the film was incubated with secondary antibodies including goat anti-mouse IgG-HRP and goat anti-rabbit IgG-HRP for 1 h at room temperature and washed again. Finally, protein bands were detected by Image Quant LAS 4000 mini (GE Healthcare).

### 2.7 Lipid extraction and analysis

Lipidomics service is provided by Shanghai Applied Protein Technology. The specific experimental steps are as follows: (1) Sample preparation and lipid extraction: lipids were extracted according to the MTBE method. Briefly, samples with equal amounts of protein were first spiked and then homogenized with 200 μL water and 240 μL methanol. After that, 800 μL of MTBE was added and the mixture was ultrasound 20 min at 4°C followed by sitting still for 30 min at room temperature. The solution was centrifuged at 14,000 × *g* for 15 min at 10°C and the upper organic solvent layer was obtained and dried under nitrogen. (2) LC-MS/MS method for lipid analysis: reverse phase chromatography was selected for LC separation using CSH C18 column (1.7 μm, 2.1 mm × 100 mm, Waters). The lipid extracts were re-dissolved in 200 μL 90% isopropanol/acetonitrile, centrifuged at 14,000 *g* for 15 min, finally, 3 μL of the sample was injected. Solvent A was acetonitrile-water (6:4, v/v) with 0.1% formic acid and 0.1 mM ammonium formate, and solvent B was acetonitrile–isopropanol (1:9, v/v) with 0.1% formic acid and 0.1 mM ammonium formate. The initial mobile phase was 40% solvent B at a flow rate of 300 μL/min. It was held for 3.5 min, and then linearly increased to 75% solvent B in 9.5 min, and then linearly increased to 99% solvent B in 6 min, followed by equilibrating at 40% solvent B for 5 min. Mass spectra was acquired by Q-Exactive Plus in positive and negative mode, respectively. ESI parameters were optimized and preset for all measurements as follows: Source temperature, 300 °C; Capillary Temp, 350 °C, the ion spray voltage was set at 3,000 V, S-Lens RF Level was set at 50% and the scan range of the instruments was set at m/z 200e temperature, 300 chromatography was selected for LC separation using CSH C18 the identification of lipid molecules based on MS/MS math. Lipid Search contains more than 30 lipid classes and more than 1,500,000 fragment ions in the database. Both mass tolerance for precursor and fragment were set to 5 ppm.

## 3 Results

### 3.1 Isolation and characterization of EVs from uninfected and EV71-infected RD cells

**FIGURE 1:**
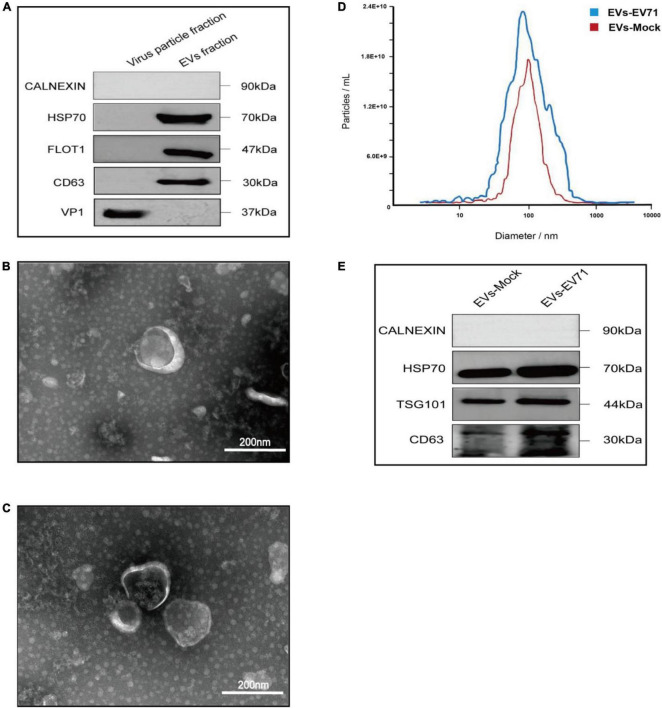
Characterization of EVs-EV71 and EVs-Mock. **(A)** WB of the components of virus particle and EVs. **(B)** Morphology of EVs-Mock observed via TEM. **(C)** Morphology of EVs-EV71 observed via TEM. **(D)** The concentration and size distribution of EVs-Mock and EVs-EV71 measured by NTA. **(D)** Particle size of EVs-EV71 measured by NTA. **(E)** WB of EV-positive markers (HSP70, TGS101 and CD63) and negative markers (Calnexin).

In this experiment, we used RD cells as cell models. Cell culture supernatants from EV71 (MOI = 1) infected cells for 24 h contained both EVs secreted by infected cells (EVs-EV71) and free EV71 virus particles. Based on the difference in particle sizes of virus particles (20–30 nm) and EVs (35–300 nm), we used differential velocity centrifugation combined with SEC method to extract EVs (EV-mock and EVs-EV71) secreted by mock-infected and EV71-infected cells, respectively. The qEV/35 nm column has high recovery for particles in the 35–350 nm range (Zamboni et al., 2022), and thus the two groups of particles (EVs and EV71 virions) can be separated relatively efficiently. WB experiments showed the EV71 structural protein VP1 was not detected in the EVs-EV71 extracts, but this does not rule out the possibility of occasional viral particles wrapped in EVs-EV71 (Figure 1A).

Then, we characterized EVs-enriched medium particle fraction (35–350 nm) by using TEM, NTA, and WB, respectively. TEM images (Figures 1B, C) and NTA measurements (Figure 1D) showed small, cup-shaped membrane vesicles with a diameter of 35–300 nm for both EVs-mock and EVs-EV71 groups. The WB analysis (Figure 1E) shows that these two groups both contained EV-positive markers (HSP70, TSG101, and CD63) but were depleted of the endoplasmic reticulum protein, Calnexin. Together, these results indicated that the purified EV products in the obtained extracts were consistent with previously described the morphology and sizes of EVs.

Based on analysis of EVs samples derived from an equal number of cell supernatants (8 × 10^7^ RD cells), the quantity of EVs increased following infection with EV71, from 5 × 10^11^ EV particles/ml to 7.4 × 10^11^ EV particles/ml (Figure 1D). Additionally, total protein quantification results showed that the protein content of the EVs-mock group was 1,650 μg/mL, and that of the EVs-EV71 group was 2,400 μg/mL. The characterization of EVs revealed that the relative expression of EV-positive markers in the EVs-EV71 group was elevated compared to the EVs-Mock group (Figure 1E). This aligns with previous studies indicating that EV71 infection enhances the secretion of EVs (Fu et al., 2017; Wu et al., 2023).

### 3.2 Lipidomic profile of EVs

International Lipid Classification and Nomenclature Committee classifies lipids into 8 categories, each of which can be subdivided into different lipid classes based on the difference in the polar head, and each of which is classified according to the degree of saturation or length of the carbon chain. Each class is divided into different lipid molecules according to the saturation of the carbon chain or the length of the carbon chain, thus constituting a three-tiered classification of lipid compounds into category, class, and molecules (Fahy et al., 2005).

**FIGURE 2:**
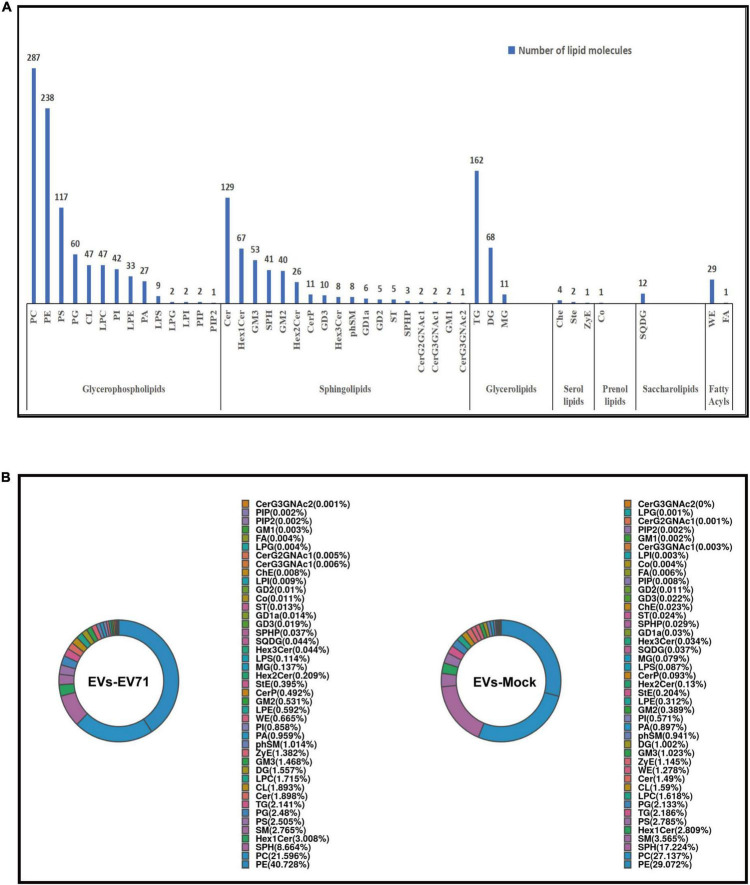
Lipidomic profile of EVs. **(A)** Number of lipid classes and molecules detected in EVs-EV71 and EVs-Mock. **(B)** Composition of lipid classes.

In this study, the lipid composition of EVs was analyzed by untargeted lipidomics based on the UPLC-Orbitrap mass spectrometry system, and a total of 7 lipid categories, 43 lipid classes, and 1,705 lipid molecules. The majority of lipid molecules (1655 lipid molecules) belong to 36 lipid classes within the three lipid categories of glycerophospholipids, sphingolipids, and glycerolipids. Among the lipid classes, PE, PC, TG, and Cer had the richest number of lipid molecules detected (Figure 2A). Lipid designations are given in Supplementary Table 1.

The lipid class composition of each group of samples is presented in a ring diagram, as shown in Figure 2B. A ring diagram corresponds to a group of samples. The lipid classes with higher proportions are the main lipid components of the samples. Analysis of the composition of lipid classes of the extracts showed that the main lipid classes of the two groups of EVs were PE, PC, and SPH. These three lipid classes were found in large quantities in the analyzed EVs, making up approximately two-thirds of the total amount of lipid detected by the test. Comparing EVs in the EVs-EV71 group to the EVs-Mock group, they revealed a 25% increase in PE (40.728 vs. 29.072%), a 20% decrease in PC (21.596 vs. 27.137%), and a 50% decrease in SPH (8.664 vs. 17.224%) (Figure 2B).

### 3.3 EV71 infection changes the lipid composition of EVs

Unlike polar metabolites such as amino acids and nucleotides, the structure of lipids is diverse, with three levels: categories, classes, and molecules, and different lipid structure levels corresponding to different lipid function levels. Therefore, the analysis of lipid content involves the overall, class and molecule levels, which helps to reveal the content differences of lipids in a more systematic and comprehensive way.

**FIGURE 3:**
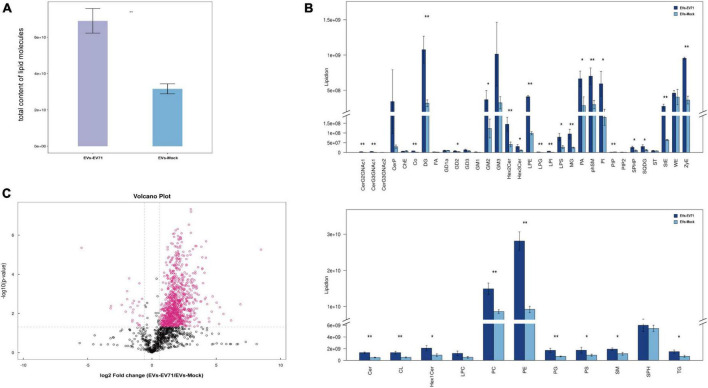
Changes of the lipid composition. **(A)** Total content of lipid molecules. **(B)** Differences in the content of lipid classes between EVs-EV71 and EVs-Mock (*P < 0.05; **P < 0.01). **(C)** Volcano plot of lipid molecules compared between EVs-EV71 and EVs-Mock.

At the overall level, we obtained the total lipid content of the two groups of EVs by adding the content of all quantified lipid molecules in the two groups of EVs. It was clearly found that the total lipid content of the EVs-EV71 group was significantly higher than that of the EVs-Mock group (Figure 3A).

At the level of lipid classes, most lipid classes showed higher expression in the EVs-EV71 group when compared to the EVs-Mock group. Particularly, the result showed that there was a considerable rise in the level of glycerophospholipids (PC, PE, PA, PI, LPE, LPS, and CL), sphingolipids (GM2, Cer, and phSM), and glycerides (DG and MG) (Figure 3B).

At the level of lipid molecules, we analyzed all the detected lipid molecules for differences based on univariate analysis such as Fold Change Analysis (FC Analysis). Lipid molecules satisfying FC > 1.5, *p* < 0.05, or FC < 0.67, *p* < 0.05 were indicated by different colors. As shown in Figure 3C, 1021 lipid molecules were significantly altered in the EVs-EV71 group compared with the EVs-Mock group, of which 996 were up-regulated and 25 were down-regulated (Supplementary Table 2).

### 3.4 Screening of significantly different lipid molecules in EVs

Through multidimensional statistical analysis, the multivariate statistical analysis method is used to reduce and classify the data. The Variable Importance for the Projection (VIP) obtained by the construction of the OPLS-DA model can be used to measure the expression patterns of various lipid molecules and the influence strength and interpretation ability of sample classification and discrimination of each group and to explore the differential lipid molecules with biological significance. Generally, lipid molecules with VIP > 1 are considered to have a significant contribution to model interpretation. In this experiment, a total of 118 significantly different lipid molecules were screened for OPLS-DA VIP > 1 and *P* < 0.05, of which 5 lipid molecules significantly decreased, and 112 lipid molecules significantly increased. Table 1 lists the top 5 lipid molecules with significant differences, and the remaining lipid molecules with significant differences are shown in Supplementary Table 3.

**TABLE 1:** Top 5 lipid molecules with significant differences (EVs-EV71 vs. EVs-Mock).

	Lipid molecules	Class	Fold change	*P*-value	VIP
Up	SPH(d18:2)+Na	SPH	37.53096778	0.034640971	1.435446456
	PG(18:1_14:0)-H	PG	21.49413602	0.01812982	1.62833616
	LPC(20:4)+H	LPC	19.59769458	0.003371336	1.071454781
	PE(38:3e)-H	PE	14.21984597	0.002457011	3.165707483
	PE(16:0p_20:1)+H	PE	11.72941751	0.043230517	7.533081504
Down	PC(30:0)+H	PC	0.02249025	4.48124E-06	15.3382151
	Cer(m38:2)+NH4	Cer	0.292726919	0.000162341	1.179107314
	PE(40:7e)+Na	PE	0.354045482	0.003071945	1.044040153
	PC(23:1_11:2)+H	PC	0.498921397	0.002603845	2.609837711
	SM(d18:0_20:4)+H	SM	0.511832757	0.038804843	1.001800824

## 4 Discussion

EVs are lipid bilayer particles, containing a wide range of molecules and metabolites, which could be transported to recipient cells for intercellular communication (van Niel et al., 2018). In recent years, it has been found that EVs can be hijacked by several non-enveloped viruses and that the association of viruses with EVs provides a number of advantages for their propagation, such as their release via non-cleavage-energy pathways, which preserves infected virus-producing cells, the possibility that viral proteins are not interfered with by neutralizing antibodies in the host organism or the possibility of spreading via wider transmission routes (Nieto-Garai et al., 2022). EV71 has been endemic globally for many years, seriously threatening the health and safety of children, the pathogenic mechanism of EV71 infection in host cells still needs to be further investigated from different perspectives. EV71 could significantly increase the secretion of EVs from host cells and EVs can be used as a vector for the spread of EV71 between cells so that the virus can escape immune surveillance and further spread in uninfected cells (Fu et al., 2017; Gu et al., 2020a,b; Wang et al., 2020; Ruan et al., 2022). Virus-induced remodeling of host cell lipid metabolism is a prominent feature of viral infections that affects viral entry, replication of genomic material, and release of progeny (Balgoma et al., 2020). However, the research about the lipid composition of virus-infected cells or their EVs is very limited. To fill this gap, here, we explored the lipid composition of EVs derived from EV71 infected cells using lipidomic research methods. RD cells are used as a research model due to their high susceptibility to EV71 infection and are widely applied for EV71 virus isolation, vaccine preparation, and pathogenic mechanism studies. Therefore, it is representative to study the effect of EV71 infection on the characterization and lipidomic of EVs using RD cells as model cells.

Over the past decades, various techniques have been developed to isolate and purify EVs. The traditional methods utilize the EV buoyant density by centrifugation, including ultracentrifugation, and density gradient ultracentrifugation. The method based on the fact that EVs change their solubility and/or aggregate in the specific medium is second in its popularity after ultracentrifugation, including precipitation with polyethylene glycol (PEG), protamine, and sodium acetate. Recently, EVs isolation methods based on affinity interactions with the molecules exposed on the EV surface or microfluidic devices have appeared. In addition, another method of EV isolation based on their size, includes ultrafiltration, hydrostatic dialysis, and gel filtration (Konoshenko et al., 2018). Considering that EV71 (1.18–1.26 g/cm^3^) and EVs (1.10–1.12 g/cm^3^) have similar buoyant density (Gu et al., 2020b), there is a risk of contamination with the viral particles using ultracentrifugation or density gradient centrifugation alone. Similarly, PEG precipitation makes it difficult to separate the EVs from virions due to their similar physical characteristics. Immunoaffinity purification can produce highly pure EVs populations, but it is limited by sample size and the quantity of the final product (McNamara and Dittmer, 2020). However, the expression levels of EV markers, such as CD9, CD63, and CD81, can vary depending on the source of EVs and physiological conditions. Therefore, a combination of multiple markers is necessary for accurate identification. Additionally, the current research demonstrates several protocols for EVs isolation based on EVs size, including ultrafiltration, hydrostatic dialysis, and gel filtration (SEC). Although the ultrafiltration and hydrostatic filtration dialysis both rely on the use of membranes with specified pore diameters to isolate particles of a pre-determined size range, these two techniques could not eliminate the small particles < 30 nm in diameter. Actually, many researchers have successfully separated EVs from other impurities such as viruses, protein aggregates, nucleosomes, and low-density lipoproteins (LDL) by using sucrose or iodixanol gradients in combination with ultracentrifugation based on the principle of difference in density of substances to ensure the purity of EVs (Iwai et al., 2016; Willms et al., 2016, 2018; Onódi et al., 2018). In this study, considering that the EV71 virus and EVs-EV71 are closer in buoyant density but completely different in size (Benedikter et al., 2017; Corso et al., 2017; Sidhom et al., 2020; Kaddour et al., 2021), the molecular size-based SEC method was finally chosen as the purification technique for EVs-EV71 following differential velocity centrifugation in this experiment.

SEC applies a column filled with porous polymer microspheres, where molecules pass through the microspheres according to their diameter; molecules with smaller radii take longer to migrate through the pores of the column, while larger molecules elute from the column earlier, accurately separating large and small molecules (Sidhom et al., 2020). Compared to ultracentrifugation, SEC is usually performed using gravitational flow, which is not affected by shear, so the structure and integrity of the vesicles remain largely intact and the bioactivity of the EVs is preserved (Gámez-Valero et al., 2016), which is more conducive to subsequent lipid analysis. Subsequently, utilizing LC-MS, we thoroughly investigated alterations in the lipid composition of the EVs after EV71 infection. The lipidomic analysis in this study revealed that following EV71 infection, the levels of most lipid classes in EVs derived from cells exhibited varying degrees of increase, with the total lipid content significantly higher than that of EVs derived from uninfected cells. Furthermore, a significant disparity in lipidomic profiles was observed between the two groups of EVs.

Viruses such as EV71 (Gu et al., 2020b; Nieto-Garai et al., 2022), norovirus (Santiana et al., 2018), rhinovirus (Feng et al., 2013), hepatitis A (HAV) (Feng et al., 2013), rotavirus (Santiana et al., 2018), or poliovirus (Bird et al., 2014) are able to use different cellular mechanisms for the production of EVs containing viral components involved in the entry of associated viral particles into a new host cell and in the process of viral transport. Meanwhile, from a lipidomic perspective, the viral infection could disrupt host cell lipid metabolism (Balgoma et al., 2020), subsequently leading to a change in the lipid composition in EVs. and the role played by various lipids in the effects of infection. Lipid analysis showed lipid enrichment in EVs after EV71 infection, with a significant increase in the content of many lipid classes, which may have an impact on the course of its infection in receipt cells. Previous studies showed viruses utilize pre-existing lipid signals for viral entry and transport as well as for reprogramming lipid synthesis, metabolism, and compartmentalization to facilitate assembly and outgrowth (Nieto-Garai et al., 2022). This suggests that the lipid enrichment of the EVs-EV71 may contribute to its infection efficiency in receipt cells including its bind, entry, releasing of content, and other life cycles. Nevertheless, the lipid enrichment of the EVs-EV71 could provide the energy to complete the EVs-EV71 life cycle in the receipt cells, because the lipids represent a great source (Omasta and Tomaskova, 2022). Obviously, the change of lipids composition in donor cells after EV71 infection should be studied in the follow-up. Due to the paucity of studies on specific lipids in EVs, the exact molecular mechanisms by which specific lipids play a role in EVs versus viruses remain to be elucidated, and we illustrate the potential role of lipids based on existing studies.

Recent lipidomics studies revealed that the biogenesis of EVs is a highly controlled process in which lipids play an indispensable role (Colombo et al., 2013). The lipidomic analysis revealed a notable increase in lipids crucial for the biogenesis of EVs after EV71 infection, including Cer, PA, and DG. Cer is synthesized by SM following the removal of the phosphocholine moiety (Trajkovic et al., 2008). Due to its conical structure, Cer could induce spontaneous negative curvature of the endosomal membrane surface (Stuffers et al., 2009). Similar to Cer, PA is the simplest phospholipid structure, characterized by a small headgroup and conical structure that enable it to naturally induce negative curvature in endosomal membranes (Kooijman et al., 2003, 2005). These lipids are involved in the induction of spontaneous membrane invagination and activate ESCRT-independent mechanisms that control EVs’ biogenesis (Cheng and Hill, 2022). Moreover, DG has the ability to recruit cytoplasmic proteins to the cell membrane, stabilize protein complexes present on the membrane, and activate membrane proteins, all of which contribute to facilitating vesicle budding and fusion processes (Sprong et al., 2001). Based on the results of this experiment and previous studies (Fu et al., 2017; Wu et al., 2023), EV71 infection significantly elevated the secretion of EVs by cells. These results indicate that EV71 can modify the lipid composition and impact crucial stages in the formation of EVs, probably leading to elevated secretion of EVs.

EVs possess a lipid bilayer structure, providing protection for a diverse range of encapsulated biomolecules against degradation and destruction (Kalluri and LeBleu, 2020). The stability of the lipid bilayer structure is reliant on the functional role of lipids. CL, a phospholipid with four fatty acid tails, stands out due to its larger size compared to other lipids possessing only two tails. This unique structure is responsible for maintaining the negative curvature of the inner membrane of lipid bilayers by altering the head-to-tail size ratio of membrane lipids (Haraszti et al., 2016). Unlike CL, lipids like lysophosphatidyl derivatives, which possess just one fatty acid tail, contribute to maintaining a positive curvature in the outer membrane of lipid bilayers (Haraszti et al., 2016). The preservation of the curvature of both the inner and outer membranes is essential for maintaining the stability of the overall membrane structure. Our results revealed that EV71 infection induced an EVs enrichment of lipids, including CL, LPE, and LPS, which may enhance bilayer membrane stability, thereby fostering a more stable viral transporter. However, the precise underlying mechanism remains elusive and warrants further investigation.

Serving as messengers for biological information transfer, EVs can pass biomolecules from one cell to another and facilitate the exchange of information (Li et al., 2021). Research suggests that EVs’ lipids play a role in the internalization and fate of their contents within recipient cells (Donoso-Quezada et al., 2021). PS, a phospholipid with the ability to bind TIM-1 and TIM-4 receptors in distinct cells, acts as a bridge between T-cells and antigen-presenting cells, enhancing their interactions and antigen presentation (Miyanishi et al., 2007). Some studies have found that during the infection of certain picornaviruses, there is an enrichment of PS in EVs, which play a role in viral packaging within vesicles and result in increased viral replication (Pleet et al., 2016; Chahar et al., 2018). In addition, in a study of extracellular vesicles in skeletal muscle, certain DG molecules were found to be highly abundant in EVs produced by IGF-1-treated myotubular cells, giving them specific targeting properties, interacting with sphingolipids in target tissues, and modulating signaling cascades in receptor cells (Valentino et al., 2021). The elevated levels of these lipids in EVs-EV71 could potentially influence the signaling pathways of the recipient cells, maybe modulating the infection and the immune response to the virus.

Our study also identified significant differential lipid molecules, such as SPH(d18:2)+Na and PG(18:1_14:0)-H, upon EV71 infection of RD cells. However, despite their importance, lipids in EVs have received less attention compared to other components like proteins and nucleic acids. The precise function of many lipids in EVs remains enigmatic. The findings presented in this study offer novel insights and strategies for further exploring the therapeutic mechanisms of EV71.

## 5 Conclusion

We demonstrated by mass spectrometry analysis that EV71 infection affects the lipid composition in EVs, which may be relevant to the subsequent function of EVs-EV71 using the RD cell model. Through correlation analysis, we found the variability of lipids in cellular EVs after EV71 infection. These studies provide new ideas for the role of lipid signaling in EVs-EV71-mediated viral diffusion, although the research is still limited by technical difficulties such as EV isolation and lipid validation.

## Data availability statement

The original contributions presented in the study are publicly available. This data are available at 10.6084/m9.figshare.26933296 (https://figshare.com/articles/dataset/Lipid_profiles_of_secreted_extracellular_vesicles_from_uninfected_and_EV71-infected_RD_cells/26933296?file=48994912).

## Author contributions

LM: Writing – original draft, Writing – review & editing. QinG: Writing – original draft, Writing – review & editing. YS: Writing – review & editing. CB: Writing – review & editing. HX: Writing – review & editing. QC: Writing – review & editing. QiaG: Writing – review & editing. FH: Writing – review & editing. WH: Writing – review & editing. JW: Writing – review & editing.

## Footnotes

1
http://www.izon.com
