# Supplementary material for: EV71 infection alters the lipid composition of human rhabdomyosarcoma (RD) cells-derived extracellular vesicles
Source: Front Microbiol. 2024 Sep 5;15:1430052. doi: 10.3389/fmicb.2024.1430052 (PMC11411429; doi:10.3389/fmicb.2024.1430052)
Supplement: Supplementary file 1 [file Table_1.DOCX]

**Table S1.** Description of the naming of lipid classes.

| **Lipid catgory** | **Lipid class** | **Abbreviations for lipid class** |
| --- | --- | --- |
| Glycerophospholipids | lysophosphatidylcholine | LPC |
|  | phosphatidylcholine | PC |
|  | lysophosphatidylethanolamine | LPE |
|  | phosphatidylethanolamine | PE |
|  | lysophosphatidylserine | LPS |
|  | phosphatidylserine | PS |
|  | lysophosphatidylglycerol | LPG |
|  | phosphatidylglycerol | PG |
|  | lysophosphatidylinositol | LPI |
|  | phosphatidylinositol | PI |
|  | phosphatidylinositol(4)phosphate | PIP |
|  | phosphatidylinositol(4,5)bisphosphate | PIP2 |
|  | phosphatidic acid | PA |
|  | Cardiolipin | CL |
| Sphingolipids | sphingomyelin | SM |
|  | phytosphingosine | phSM |
|  | Sphingosine | SPH |
|  | Sphingosine phosphate | SPHP |
|  | glucocerebroside /Hexosyl ceramide | CerG1/Hex1Cer |
|  | glucocerebroside/Hexosyl ceramide | CerG2/Hex2Cer |
|  | glucocerebroside/Hexosyl ceramide | CerG3/Hex3Cer |
|  | N-acetylhexosyl ceramide | CerG2GNAc1 |
|  | Dihexosyl N-acetylhexosyl ceramide | CerG3GNAc1 |
|  | Trihexosyl di-N-acetylhexosyl ceramide | CerG3GNAc2 |
|  | Sulfatide (galactosyl ceramide sulfate) | ST |
|  | Ceramides | Cer |
|  | Ceramides phosphate | CerP |
|  | Ganglioside, monosialo trihexosyl ceramide | GM3 |
|  | Ganglioside, monosialo dihexosyl ceramide | GM2 |
|  | Ganglioside, monosialo tetrahexosyl ceramide | GM1 |
|  | Ganglioside, disialo tetrahexosyl ceramide | GD1a |
|  | Ganglioside, disialo dihexosyl ceramide | GD2 |
|  | Ganglioside, disialo trihexosyl ceramide | GD3 |
| Glycerolipids | monoglyceride | MG |
|  | diglyceride | DG |
|  | triglyceride | TG |
| Serol lipids | Cholesterol Ester | ChE |
|  | zymosterol | ZyE |
|  | Stigmasterol ester | StE |
| Prenol lipids | Coenzyme Q | Co |
| Fatty Acyls | wax exters | WE |
|  | fatty acid | FA |
| Saccharolipids | Sulfoquinovosyldiacylglycerol | SQDG |

**Table S2.** Significantly different lipid molecules analysed by univariate statistics.

| DOWN(EVs-EV71 vs EVs-Mock) |  |  |  |
| --- | --- | --- | --- |
| **Lipid molecules** | **Class** | **Fold Change** | **P-value** |
| SPH(t16:1)+H | SPH | 0.610360503 | 0.01364542 |
| PC(36:5)+H | PC | 0.589300695 | 0.001817495 |
| PS(18:3e_20:5)-H | PS | 0.51847592 | 0.0128963 |
| DG(17:1e)+NH4 | DG | 0.514095437 | 0.042336451 |
| SM(d18:0_20:4)+H | SM | 0.511832757 | 0.038804843 |
| PS(41:1)+Na | PS | 0.507356878 | 0.000288018 |
| PC(23:1_11:2)+H | PC | 0.498921397 | 0.002603845 |
| TG(15:0_14:0_16:1)+NH4 | TG | 0.479699552 | 0.037941021 |
| ChE(20:4)+NH4 | ChE | 0.460066913 | 0.004108243 |
| TG(15:0_16:0_24:0)+NH4 | TG | 0.453624867 | 0.031354401 |
| ST(t38:6)+HCOO | ST | 0.3777627 | 0.002316636 |
| PIP(12:0e_21:1)-H | PIP | 0.366774925 | 0.006386265 |
| PE(40:7e)+Na | PE | 0.354045482 | 0.003071945 |
| SPH(d19:1)+H | SPH | 0.353026141 | 0.008947492 |
| TG(24:2_10:3_11:1)+H | TG | 0.351819355 | 0.021758068 |
| ST(d37:3+2O)-H | ST | 0.346795326 | 0.026158624 |
| GD1a(m39:1)-2H | GD1a | 0.315674602 | 0.004864116 |
| Cer(m38:2)+NH4 | Cer | 0.292726919 | 0.000162341 |
| PE(38:6e)+Na | PE | 0.263945805 | 0.001891942 |
| PC(40:9)+H | PC | 0.263801742 | 0.00073012 |
| DG(42:6)+NH4 | DG | 0.244081773 | 0.008115301 |
| PS(16:2e_20:3)-H | PS | 0.196502442 | 0.003697184 |
| SPH(d24:2)+H | SPH | 0.110261419 | 0.004227997 |
| GM3(m40:3)+H-H2O | GM3 | 0.107792796 | 0.005522994 |
| PC(30:0)+H | PC | 0.02249025 | 4.48124E-06 |
|  |  |  |  |
| UP(EVs-EV71 vs EVs-Mock) |  |  |  |
| **Lipid molecules** | **Class** | **Fold Change** | **P-value** |
| DG(31:2e)+H | DG | 357.9061895 | 5.54804E-06 |
| DG(30:2e)+H | DG | 118.2160475 | 0.003442588 |
| PG(16:0_14:0)-H | PG | 71.57161862 | 0.02305639 |
| SQDG(40:5)-H | SQDG | 47.18824949 | 0.010661945 |
| PG(15:0_16:0)-H | PG | 38.86910609 | 0.004887138 |
| SPH(d18:2)+Na | SPH | 37.53096778 | 0.034640971 |
| PG(17:1_16:1)-H | PG | 32.60604906 | 0.002859603 |
| PC(16:1_20:5)+H | PC | 26.40823882 | 0.001365663 |
| PG(15:0_15:0)+H | PG | 25.03833036 | 0.008120954 |
| PC(16:1_20:5)+H | PC | 21.98612224 | 0.026417196 |
| PG(18:1_14:0)-H | PG | 21.49413602 | 0.01812982 |
| LPC(20:4)+H | LPC | 19.59769458 | 0.003371336 |
| Cer(d36:2)+HCOO | Cer | 18.54026585 | 2.09423E-06 |
| LPG(16:0)-H | LPG | 18.32725836 | 1.4318E-05 |
| PI(16:0_18:3)-H | PI | 18.14153862 | 0.001582419 |
| PG(16:1_18:2)-H | PG | 17.42182722 | 0.001901707 |
| PE(30:2e)-H | PE | 16.99033116 | 8.31812E-05 |
| Hex2Cer(d34:2)+HCOO | Hex2Cer | 15.78692158 | 0.000282599 |
| PI(18:0_18:0)+Na | PI | 15.15291285 | 4.98798E-05 |
| LPC(20:4e)+H | LPC | 15.06471758 | 5.39501E-05 |
| WE(2:0_23:3)+NH4 | WE | 15.06183174 | 1.37007E-05 |
| WE(2:0_25:3)+NH4 | WE | 14.36077556 | 3.95607E-06 |
| PE(38:3e)-H | PE | 14.21984597 | 0.002457011 |
| Hex2Cer(d40:2)+HCOO | Hex2Cer | 13.63239845 | 0.002511874 |
| PC(16:0e_14:1)+HCOO | PC | 13.2363388 | 0.028490863 |
| WE(2:0_25:3)+NH4 | WE | 12.38039342 | 0.001141971 |
| WE(22:3)+NH4 | WE | 12.34884801 | 0.027763504 |
| LPC(18:3e)+H | LPC | 12.0769688 | 4.36065E-05 |
| PE(16:0p_20:1)+H | PE | 11.72941751 | 0.043230517 |
| WE(8:0_17:3)+NH4 | WE | 11.40251133 | 0.005695078 |
| PS(22:0)-H | PS | 11.3159995 | 0.000471598 |
| PE(41:3e)+Na | PE | 11.27180062 | 0.000983936 |
| PC(38:4e)+H | PC | 11.08297029 | 0.018159378 |
| LPC(18:1e)+HCOO | LPC | 11.03481738 | 0.000538319 |
| PC(34:4)+H | PC | 11.02403783 | 0.002398718 |
| LPC(18:1e)+HCOO | LPC | 10.98303437 | 3.65744E-06 |
| PG(18:1_22:6)-H | PG | 10.91072483 | 0.00038175 |
| LPG(18:1)-H | LPG | 10.42114043 | 3.62729E-06 |
| CerG3GNAc2(d34:2+O)+H | CerG3GNAc2 | 10.38970598 | 9.14112E-05 |
| PG(15:0_15:0)-H | PG | 10.38046346 | 7.37084E-05 |
| PC(42:10)+H | PC | 10.25024042 | 0.009919218 |
| TG(42:11)+H | TG | 10.23992068 | 0.002057409 |
| PG(16:0_22:6)-H | PG | 10.20872159 | 0.000448712 |
| WE(2:0_16:2)+NH4 | WE | 10.16747375 | 0.00187772 |
| TG(40:10e)+Na | TG | 10.0260819 | 0.000889792 |
| Hex2Cer(t34:0)+HCOO | Hex2Cer | 9.977017019 | 0.00567107 |
| PC(32:2e)+HCOO | PC | 9.893330232 | 0.021308588 |
| DG(38:5e)+H | DG | 9.891131019 | 0.000200108 |
| GM1(d48:2+O)+HCOO | GM1 | 9.887690136 | 0.047870183 |
| ST(t40:6)+NH4 | ST | 9.719854673 | 0.013032459 |
| PG(16:1_16:1)-H | PG | 9.583951025 | 0.009534359 |
| DG(12:0e_24:2)+Na | DG | 9.513713506 | 0.000373557 |
| Hex2Cer(d32:1)+HCOO | Hex2Cer | 9.436979973 | 0.007436187 |
| GD2(d34:1)-2H | GD2 | 9.424087947 | 0.003106527 |
| Cer(m18:0_22:1)+H | Cer | 9.328136299 | 0.000375065 |
| TG(44:12)+H | TG | 9.250590599 | 0.000503722 |
| GD3(d34:0)-2H | GD3 | 9.218181613 | 0.002638782 |
| PC(34:4)+H | PC | 9.174387356 | 8.78466E-05 |
| PE(18:0_18:0)+Na | PE | 9.137321386 | 5.28342E-06 |
| GD3(d34:1)-2H | GD3 | 8.938858366 | 0.000375453 |
| DG(35:0)+H | DG | 8.882697278 | 0.000239036 |
| LPC(18:1)+HCOO | LPC | 8.850670646 | 0.000170621 |
| LPC(16:0e)+HCOO | LPC | 8.740844847 | 0.00152271 |
| PI(18:1_20:5)-H | PI | 8.648960454 | 0.014058544 |
| PI(18:0_18:3)-H | PI | 8.576576127 | 0.00615994 |
| LPC(16:1)+HCOO | LPC | 8.476121977 | 0.000279397 |
| PE(18:0_16:0)+Na | PE | 8.465860549 | 0.001598816 |
| PG(40:8)-H | PG | 8.447583414 | 0.001133174 |
| PC(33:3)+H | PC | 8.406928488 | 0.000688665 |
| LPC(14:0)+HCOO | LPC | 8.254704542 | 6.36645E-08 |
| DG(32:2e)+H | DG | 8.234963664 | 0.010148727 |
| DG(18:1_22:1)+Na | DG | 8.224361704 | 0.0146149 |
| CerG2GNAc1(d34:1)+H | CerG2GNAc1 | 8.212931265 | 0.000192803 |
| PG(16:1_16:1)-H | PG | 8.173825776 | 0.007122915 |
| PG(18:1_18:3)-H | PG | 8.173316466 | 7.60643E-07 |
| PE(16:0p_20:3)+H | PE | 8.146676461 | 4.81465E-08 |
| PI(18:0_20:3)-H | PI | 8.12470395 | 0.005929603 |
| PE(18:1_22:3)+H | PE | 8.121734361 | 0.00732164 |
| LPC(28:1)+H | LPC | 8.030774983 | 0.000244057 |
| LPC(16:1e)+HCOO | LPC | 7.991777089 | 1.00626E-05 |
| PI(17:0_20:3)-H | PI | 7.938026831 | 0.012013459 |
| SM(d18:1_23:4)+H | SM | 7.917728283 | 0.007869995 |
| Hex3Cer(d32:1)+HCOO | Hex3Cer | 7.880234754 | 0.026765599 |
| PG(16:1_18:1)-H | PG | 7.839780804 | 0.015460707 |
| Cer(d44:3)+HCOO | Cer | 7.835118273 | 0.042961328 |
| CL(18:1_16:1_18:1_18:1)-H | CL | 7.783439532 | 0.013890102 |
| SQDG(39:5)-H | SQDG | 7.768795704 | 0.001610817 |
| PC(36:5e)+H | PC | 7.764755383 | 0.001265691 |
| PS(35:2e)+H | PS | 7.706487068 | 0.006993413 |
| PA(20:4e_24:2)-H | PA | 7.696248863 | 0.000118949 |
| LPC(20:2)+H | LPC | 7.694861109 | 0.000564474 |
| LPC(18:0e)+HCOO | LPC | 7.63040648 | 6.73781E-06 |
| PI(18:1_18:3)-H | PI | 7.618062384 | 0.029357741 |
| SPH(t20:1)+H-H2O | SPH | 7.613867287 | 0.016081264 |
| GM2(m36:2)+H | GM2 | 7.508390047 | 0.002886839 |
| LPE(18:1e)-H | LPE | 7.498093839 | 0.007622001 |
| PG(16:1_18:2)-H | PG | 7.471409072 | 0.007066109 |
| PG(15:0_16:1)-H | PG | 7.468730812 | 0.003315603 |
| PG(16:0_16:1)-H | PG | 7.35849066 | 0.000892704 |
| PC(10:1e_6:0)+H | PC | 7.335791681 | 2.92877E-07 |
| PG(18:1_22:5)-H | PG | 7.25304944 | 0.005894064 |
| PG(18:1_20:4)-H | PG | 7.247658025 | 0.001113802 |
| TG(42:11e)+Na | TG | 7.238752747 | 0.00039983 |
| PC(40:3e)+H | PC | 7.153913231 | 0.001586488 |
| DG(18:0_20:3)+NH4 | DG | 7.113022283 | 0.014166218 |
| PC(32:4)+H | PC | 7.088687787 | 0.006160656 |
| PC(40:3)+H | PC | 7.010279641 | 0.000180819 |
| TG(8:0_11:2_24:0)+H | TG | 6.958337088 | 0.026967896 |
| SM(d33:2)+H | SM | 6.931163888 | 0.001578071 |
| LPC(26:0)+H | LPC | 6.930956997 | 0.017328421 |
| SM(d37:1)+Na | SM | 6.907639131 | 0.007197136 |
| TG(12:0e_6:0_22:6)+H | TG | 6.874530713 | 0.018849323 |
| PC(12:0_14:0)-CH3 | PC | 6.862569924 | 0.007202421 |
| DG(34:1e)+H | DG | 6.856548826 | 0.00101127 |
| GM3(m38:2)-2H | GM3 | 6.813633624 | 0.000264743 |
| LPE(20:1)-H | LPE | 6.801252029 | 0.043806102 |
| Cer(m19:0_21:3)+H | Cer | 6.797056223 | 1.1006E-05 |
| PG(15:0_17:0)-H | PG | 6.765092736 | 0.000206044 |
| SM(d39:1)+Na | SM | 6.746796374 | 0.002342298 |
| PC(40:6e)+H | PC | 6.695408783 | 0.011963519 |
| TG(42:9)+Na | TG | 6.692900999 | 0.00074258 |
| Cer(m19:0_21:3)+H | Cer | 6.579990911 | 0.002491155 |
| PE(16:0_14:0)-H | PE | 6.546344607 | 0.001507298 |
| DG(12:0e_22:3)+Na | DG | 6.545929178 | 0.025341552 |
| Hex2Cer(m19:0_23:3)+H | Hex2Cer | 6.543603084 | 0.001524571 |
| MG(32:0)+H | MG | 6.523305354 | 0.002317611 |
| DG(32:0e)+H | DG | 6.500519214 | 0.00232832 |
| PC(8:1e_10:0)+H | PC | 6.495963289 | 9.51837E-05 |
| Co(Q10)+NH4 | Co | 6.448673867 | 3.20246E-05 |
| LPC(26:1)+H | LPC | 6.44401262 | 0.015121103 |
| PE(18:0p_22:3)+H | PE | 6.437897464 | 0.000370873 |
| PC(40:4e)+H | PC | 6.429621159 | 0.000284519 |
| CerG2GNAc1(d36:3)-2H | CerG2GNAc1 | 6.415174362 | 1.67494E-05 |
| LPC(20:1)+HCOO | LPC | 6.404839883 | 3.50496E-05 |
| PE(10:0_11:3)-H | PE | 6.29915931 | 0.001926169 |
| GM2(d38:2)-H | GM2 | 6.29270807 | 0.044196119 |
| Cer(d18:1_22:1)+HCOO | Cer | 6.271175926 | 0.018367644 |
| PC(40:6e)+H | PC | 6.231758087 | 0.002783739 |
| GM2(d37:5)-H | GM2 | 6.229512868 | 0.046790059 |
| Cer(d12:0_24:2)+HCOO | Cer | 6.220908487 | 0.001864943 |
| CL(18:1_16:1_16:1_18:1)-H | CL | 6.213710926 | 0.003740077 |
| DG(38:3e)+H | DG | 6.18706086 | 0.026417275 |
| MG(34:0)+H | MG | 6.129554729 | 0.000355837 |
| Hex2Cer(m40:0+O)+HCOO | Hex2Cer | 6.06394697 | 0.006651055 |
| TG(12:0e_6:0_22:5)+H | TG | 6.057722698 | 0.001004556 |
| Hex1Cer(m19:0_21:3)+H | Hex1Cer | 5.993965909 | 0.001131173 |
| MG(34:1)+Na | MG | 5.990819615 | 0.040587795 |
| PC(42:7e)-CH3 | PC | 5.954607652 | 0.036560284 |
| PC(8:1e_12:0)+Na | PC | 5.927451536 | 0.005997117 |
| LPC(22:4)+H | LPC | 5.927002731 | 0.00598083 |
| LPI(18:1)-H | LPI | 5.887315782 | 0.000383956 |
| PA(25:0_22:6)-H | PA | 5.886242949 | 0.016229845 |
| Hex2Cer(d40:1)+HCOO | Hex2Cer | 5.870943407 | 0.00538467 |
| PE(22:3e)+H | PE | 5.861812592 | 0.000572838 |
| PC(16:1_18:2)+HCOO | PC | 5.852018488 | 0.043560499 |
| PI(16:1_18:1)-H | PI | 5.834158532 | 0.021598133 |
| LPI(18:0)-H | LPI | 5.824069449 | 7.97682E-05 |
| PC(15:0_20:5)+H | PC | 5.78501588 | 0.00111477 |
| PE(18:1_18:1)+Na | PE | 5.774766909 | 0.000974736 |
| CL(18:2_18:1_18:1_16:1)-H | CL | 5.766929176 | 0.027085372 |
| SM(d41:2)+Na | SM | 5.715379063 | 0.006568743 |
| PS(18:1e_16:1)+H | PS | 5.708082022 | 0.003212053 |
| LPC(16:0)+HCOO | LPC | 5.707636793 | 0.000134436 |
| Hex2Cer(m19:0_23:2)+H | Hex2Cer | 5.701399165 | 0.00136752 |
| TG(22:6_10:3_11:1)+NH4 | TG | 5.689313207 | 0.003096092 |
| LPC(38:1)+Na | LPC | 5.688105731 | 0.00636519 |
| PE(12:0p_23:1)+H | PE | 5.678760918 | 0.00062171 |
| LPE(20:3)-H | LPE | 5.63742638 | 1.13167E-05 |
| LPC(15:0)+Na | LPC | 5.629948311 | 0.00616499 |
| LPE(18:1)-H | LPE | 5.621809989 | 0.000596034 |
| LPE(22:3)-H | LPE | 5.583903554 | 0.01312462 |
| TG(46:11e)+Na | TG | 5.497063005 | 0.011111722 |
| LPC(36:0)+Na | LPC | 5.486607531 | 0.026834222 |
| PE(41:2e)+H | PE | 5.451693202 | 0.000972607 |
| PE(16:1e_20:3)-H | PE | 5.445918348 | 0.001010389 |
| PE(16:1e_18:3)-H | PE | 5.432072209 | 0.004581107 |
| PC(42:3)+H | PC | 5.429722628 | 7.11989E-06 |
| PE(41:2e)+H | PE | 5.427351369 | 0.030317054 |
| PE(18:0_18:1)+Na | PE | 5.419363272 | 0.000488165 |
| PE(40:2e)-H | PE | 5.410514078 | 0.037692179 |
| PE(18:1_22:3)-H | PE | 5.410191132 | 0.024613683 |
| TG(39:2)+H | TG | 5.38088724 | 0.01388821 |
| Hex2Cer(d44:2)+HCOO | Hex2Cer | 5.377667784 | 0.013827475 |
| LPC(34:1)+Na | LPC | 5.36652722 | 0.000197025 |
| PG(16:0_16:0)-H | PG | 5.36537332 | 0.025343442 |
| Hex1Cer(m19:0_21:2)+H | Hex1Cer | 5.363062467 | 0.001527414 |
| PE(12:0p_23:0)+H | PE | 5.347922896 | 0.006257209 |
| LPC(17:0)+HCOO | LPC | 5.344732567 | 0.001261949 |
| TG(47:6e)+H | TG | 5.336483187 | 0.002376762 |
| PE(16:1p_6:0)+H | PE | 5.270142503 | 0.000145578 |
| PE(22:2e)+H | PE | 5.254638794 | 0.000106454 |
| Cer(t18:0_14:0)+H-H2O | Cer | 5.248548042 | 0.013860242 |
| CerG3GNAc1(d34:1)+H | CerG3GNAc1 | 5.239586804 | 0.000685223 |
| PC(38:7)+H | PC | 5.22235447 | 0.013794843 |
| Cer(m19:0_21:2)+H | Cer | 5.214689364 | 0.001775972 |
| PI(16:0_16:1)-H | PI | 5.21404056 | 0.022973745 |
| LPC(28:0)+H | LPC | 5.209418875 | 0.000351276 |
| PC(40:3e)+H | PC | 5.198363434 | 0.00106556 |
| Hex1Cer(d34:3)+H | Hex1Cer | 5.189567131 | 0.036644679 |
| PG(18:1_20:2)-H | PG | 5.188447514 | 0.034793646 |
| PI(18:1_14:1)-H | PI | 5.177214148 | 0.001873437 |
| Hex2Cer(d40:1)+HCOO | Hex2Cer | 5.177071309 | 0.000813853 |
| DG(36:5e)+H | DG | 5.175457854 | 1.07626E-05 |
| CerG3GNAc1(m31:1)+H-H2O | CerG3GNAc1 | 5.147799923 | 0.000251539 |
| LPC(24:0)+H | LPC | 5.147194616 | 0.020124756 |
| Cer(d18:0_22:1)+H | Cer | 5.145615878 | 0.000194621 |
| LPS(20:3)-H | LPS | 5.138660659 | 3.16808E-05 |
| PE(16:0p_14:0)+H | PE | 5.12429571 | 0.000161592 |
| LPE(22:0)-H | LPE | 5.105850972 | 0.00011011 |
| TG(16:0e_16:0_16:0)+NH4 | TG | 5.099352452 | 0.000342681 |
| SM(d36:1)+H | SM | 5.090929427 | 0.034272478 |
| SM(d34:4)+H | SM | 5.08004994 | 0.012687062 |
| LPE(16:0e)-H | LPE | 5.00732581 | 2.28981E-06 |
| PS(18:1e_18:1)+H | PS | 5.006415283 | 9.39459E-05 |
| SM(d18:2_16:1)+H | SM | 5.00640499 | 0.008135258 |
| TG(14:0e_6:0_15:0)+H | TG | 4.998664238 | 0.002059392 |
| PI(16:0_20:4)-H | PI | 4.993689544 | 0.023856503 |
| PI(18:1_18:1)+Na | PI | 4.99074303 | 0.003534978 |
| TG(18:1_10:4_14:3)+Na | TG | 4.988568471 | 0.006596722 |
| PE(35:2e)+H | PE | 4.981545361 | 0.000577944 |
| TG(6:0_11:1_24:0)+H | TG | 4.979433451 | 0.022524469 |
| PE(18:1p_22:2)+H | PE | 4.956632154 | 0.000558808 |
| LPE(16:1)-H | LPE | 4.942226232 | 8.06643E-07 |
| PC(14:1e_22:1)+H | PC | 4.94179501 | 0.037710393 |
| PC(44:2e)+HCOO | PC | 4.939089498 | 0.013749272 |
| PC(44:3e)+H | PC | 4.938955829 | 0.030440058 |
| SM(d18:1_13:0)+H | SM | 4.932487946 | 0.000323294 |
| PI(18:0_18:0)-H | PI | 4.93196898 | 0.009140616 |
| PC(29:0)+H | PC | 4.925615958 | 0.000113595 |
| SM(d44:4)+H | SM | 4.922058058 | 0.03421791 |
| PE(18:1e_22:3)-H | PE | 4.901536044 | 0.003505334 |
| TG(20:4e_11:3_11:3)+Na | TG | 4.890099757 | 0.028566578 |
| DG(30:1e)+Na | DG | 4.883708785 | 0.003997725 |
| LPE(18:0e)-H | LPE | 4.873279101 | 4.02079E-05 |
| TG(22:6_11:2_11:3)+H | TG | 4.865348533 | 0.011547584 |
| GM2(m33:0)+HCOO | GM2 | 4.842947024 | 0.014281078 |
| PC(38:3e)+H | PC | 4.823341731 | 0.000285199 |
| PC(16:1_16:1)+HCOO | PC | 4.822342544 | 0.003673053 |
| TG(16:0e_14:0_16:0)+NH4 | TG | 4.811624502 | 0.000445995 |
| PC(12:1e_18:1)+HCOO | PC | 4.806695458 | 0.004307541 |
| GM3(m36:2)+H | GM3 | 4.798572666 | 0.023540144 |
| PG(16:1_20:4)-H | PG | 4.777818839 | 0.004040494 |
| DG(34:4e)+H | DG | 4.776290542 | 0.000433381 |
| PE(16:0p_22:3)+H | PE | 4.765682274 | 0.008273405 |
| PG(18:1_22:4)-H | PG | 4.760692716 | 0.03019607 |
| GM3(t34:0)+H | GM3 | 4.75459471 | 1.58159E-06 |
| PE(40:4p)+Na | PE | 4.749646163 | 0.006916869 |
| Cer(d36:1)+H-H2O | Cer | 4.743445569 | 0.028132592 |
| GM3(d36:5)+HCOO | GM3 | 4.729228576 | 0.023412061 |
| LPE(18:2e)-H | LPE | 4.728452261 | 1.49318E-06 |
| PC(14:0_14:1)+HCOO | PC | 4.719599484 | 0.001979007 |
| PG(20:1_18:1)-H | PG | 4.703052349 | 0.004995618 |
| TG(44:9e)+NH4 | TG | 4.688895514 | 0.019341737 |
| PE(18:1e_20:3)-H | PE | 4.683227847 | 0.019876125 |
| PC(36:0e)+H | PC | 4.674184237 | 1.30148E-06 |
| PE(16:0p_22:2)+H | PE | 4.662895264 | 0.024749415 |
| MG(18:3e)+H | MG | 4.659633276 | 0.004656293 |
| PC(29:2)+H | PC | 4.651796905 | 0.00292212 |
| LPE(20:2)-H | LPE | 4.645905052 | 1.62235E-05 |
| PE(40:1e)-H | PE | 4.637915639 | 0.017087763 |
| PE(16:1e_14:0)-H | PE | 4.631095936 | 0.001216749 |
| TG(12:0e_10:4_18:0)+H | TG | 4.625553545 | 0.014642617 |
| DG(32:1e)+Na | DG | 4.619821506 | 0.000915933 |
| Hex1Cer(d32:0)+HCOO | Hex1Cer | 4.619320023 | 0.01775822 |
| TG(16:0e_16:0_18:3)+H | TG | 4.614864569 | 0.002268013 |
| LPE(16:0)-H | LPE | 4.597172784 | 3.58643E-06 |
| Cer(t18:0_18:0)+H-H2O | Cer | 4.586904381 | 0.001756827 |
| TG(12:1e_13:0_13:0)+Na | TG | 4.585151983 | 0.017109907 |
| Hex2Cer(d36:1)+HCOO | Hex2Cer | 4.580211967 | 0.011970854 |
| Hex1Cer(m32:0+O)+HCOO | Hex1Cer | 4.577827686 | 0.017911727 |
| Hex2Cer(d42:1)+HCOO | Hex2Cer | 4.571434767 | 0.012148895 |
| Cer(d32:1)+H-H2O | Cer | 4.568360871 | 2.09568E-05 |
| PC(36:0e)-CH3 | PC | 4.561674148 | 0.019127034 |
| WE(2:0_16:4)+NH4 | WE | 4.559855799 | 0.011736112 |
| PI(18:0_22:2)-H | PI | 4.559734107 | 0.032121878 |
| PG(16:0_18:1)-H | PG | 4.557935042 | 0.030116049 |
| TG(20:4e_10:2_10:3)+Na | TG | 4.55672765 | 0.000274726 |
| Cer(m19:0_23:3)+H | Cer | 4.548900797 | 1.26197E-06 |
| GD2(t44:3)-2H | GD2 | 4.547775835 | 0.002574719 |
| LPE(14:0)-H | LPE | 4.54072414 | 4.36521E-06 |
| PC(42:2e)+HCOO | PC | 4.538636343 | 0.002466315 |
| GM2(d34:1)-H | GM2 | 4.52917393 | 0.023204368 |
| PE(16:1e_20:0)-H | PE | 4.527279867 | 0.002191872 |
| Hex1Cer(d18:1_18:0)+H | Hex1Cer | 4.524946396 | 0.015540272 |
| PE(18:0p_22:6)+H | PE | 4.501082428 | 0.020401382 |
| LPC(15:0)+HCOO | LPC | 4.496144334 | 0.002475554 |
| PG(18:1_18:1)-H | PG | 4.494322794 | 0.008448222 |
| TG(18:0e_14:1_16:1)+NH4 | TG | 4.49241581 | 0.002344235 |
| PC(39:5)+H | PC | 4.492324578 | 0.009543345 |
| GM2(d34:4)+HCOO | GM2 | 4.484977907 | 0.036534995 |
| PS(42:6e)-H | PS | 4.438850305 | 0.007077799 |
| Hex2Cer(d42:1)+HCOO | Hex2Cer | 4.435637257 | 0.019212792 |
| TG(18:2_12:4_14:3)+Na | TG | 4.429058417 | 0.003592273 |
| PE(18:1p_22:6)+H | PE | 4.428324286 | 6.00087E-05 |
| PE(8:0_11:2)-H | PE | 4.426103669 | 0.000163557 |
| PE(16:1_14:0)-H | PE | 4.425435319 | 0.00218589 |
| SM(d44:5)+H | SM | 4.416632308 | 0.017031816 |
| PE(10:0p_6:0)+H | PE | 4.392371102 | 0.000209805 |
| LPC(18:2)+HCOO | LPC | 4.390998805 | 2.52309E-05 |
| Cer(m19:0_21:2)+H | Cer | 4.385300706 | 5.26884E-05 |
| MG(30:1)+Na | MG | 4.372153767 | 0.000670939 |
| PI(18:0_22:4)-H | PI | 4.371102248 | 0.017509194 |
| Cer(t18:0_24:1)+H-H2O | Cer | 4.367275422 | 0.004401369 |
| PE(8:1e_12:2)+Na | PE | 4.360080117 | 1.6225E-05 |
| CL(78:3)-2H | CL | 4.351042296 | 0.020998839 |
| Cer(m38:2)+Na | Cer | 4.350592909 | 0.037519811 |
| Cer(m19:1_21:4)+H | Cer | 4.349862322 | 0.037479034 |
| GM3(d34:4)+HCOO | GM3 | 4.347265116 | 0.028791007 |
| Hex1Cer(t34:0+O)+HCOO | Hex1Cer | 4.327615839 | 0.014691935 |
| PC(15:0_22:5)+H | PC | 4.321219731 | 0.025027122 |
| PC(35:1)+H | PC | 4.316204523 | 1.63758E-05 |
| GD2(m34:0+O)-2H | GD2 | 4.314503926 | 0.000492394 |
| Hex1Cer(d18:1_14:0)+H | Hex1Cer | 4.313394863 | 0.000467789 |
| CL(18:0_18:0_18:1_18:1)-H | CL | 4.306361405 | 0.014097671 |
| SQDG(41:5)-H | SQDG | 4.298980741 | 0.038828323 |
| PG(14:0_14:0)-H | PG | 4.294473709 | 0.015297864 |
| Cer(m36:1+O)+HCOO | Cer | 4.292389353 | 0.000404523 |
| PC(16:1_18:2)+HCOO | PC | 4.284460068 | 0.014394279 |
| Cer(d18:1_18:0)+HCOO | Cer | 4.282047735 | 0.000184061 |
| LPE(20:0)-H | LPE | 4.273983843 | 1.67948E-05 |
| PC(20:0_11:2)+H | PC | 4.271782745 | 1.78306E-06 |
| PG(16:1_14:0)-H | PG | 4.266314117 | 0.002309833 |
| LPC(22:1)+H | LPC | 4.263862715 | 1.12835E-05 |
| PG(18:0_18:1)-H | PG | 4.255561134 | 0.020230763 |
| TG(16:0e_14:0_16:1)+NH4 | TG | 4.245886075 | 0.001478217 |
| PC(38:3)+H | PC | 4.245828804 | 0.006540317 |
| PC(18:1_14:0)+H | PC | 4.237209807 | 0.002441539 |
| LPE(20:2e)-H | LPE | 4.22629283 | 9.42508E-06 |
| LPE(18:0)-H | LPE | 4.225680763 | 8.94781E-06 |
| CL(68:5)-H | CL | 4.220933279 | 0.04001332 |
| Cer(d18:1_24:2)+HCOO | Cer | 4.220282202 | 0.000845628 |
| DG(36:4e)+Na | DG | 4.217228224 | 0.012286445 |
| PS(18:0_18:1)+Na | PS | 4.216217498 | 0.003213535 |
| TG(18:0e_16:0_16:0)+NH4 | TG | 4.216024919 | 3.38016E-05 |
| Cer(t18:0_16:0)+H | Cer | 4.20497445 | 0.007669853 |
| PA(20:0_22:6)-H | PA | 4.202751234 | 0.015733033 |
| Cer(m19:0_23:3)+H | Cer | 4.199621169 | 0.005091865 |
| Cer(d34:2)+HCOO | Cer | 4.199183905 | 0.002163215 |
| Cer(d18:1_18:0)+H | Cer | 4.189194385 | 0.00057827 |
| PE(18:0e)+H | PE | 4.185488472 | 1.57116E-06 |
| PE(18:2e_20:1)-H | PE | 4.158695746 | 0.025301015 |
| Cer(t18:0_22:0)+H-H2O | Cer | 4.157106195 | 0.002917698 |
| PC(36:5e)+H | PC | 4.153138349 | 0.005975197 |
| phSM(t37:1)+H | phSM | 4.146229725 | 0.002045386 |
| PC(16:0_14:1)+HCOO | PC | 4.116403431 | 0.033915455 |
| PA(37:0)+NH4 | PA | 4.106641231 | 0.026466628 |
| PE(18:0p_16:0)+H | PE | 4.105866445 | 0.033243491 |
| Hex2Cer(m36:0+O)+HCOO | Hex2Cer | 4.102371325 | 0.011618049 |
| PG(18:1_18:2)-H | PG | 4.092603973 | 0.019231357 |
| Hex2Cer(d42:2)-H | Hex2Cer | 4.087734579 | 0.009472712 |
| PE(20:1e)+H | PE | 4.084916508 | 0.000764075 |
| PC(35:0)+H | PC | 4.082722647 | 0.003052258 |
| LPC(15:0)+H | LPC | 4.075000305 | 6.68768E-06 |
| PE(16:1e)+H | PE | 4.074420081 | 0.000460135 |
| PG(18:1_18:2)-H | PG | 4.074249783 | 0.0158102 |
| PE(18:1e_22:3)-H | PE | 4.072872568 | 0.008342726 |
| PE(18:1_20:4)-H | PE | 4.070267223 | 0.002295779 |
| PG(34:4)-H | PG | 4.066929455 | 0.046072083 |
| PC(17:1_18:1)+H | PC | 4.066325349 | 1.10665E-06 |
| Cer(d18:0_22:1)+H | Cer | 4.065772362 | 0.008499755 |
| TG(20:1e_10:0_10:0)+Na | TG | 4.063427325 | 0.042072084 |
| PE(16:0p_16:1)+H | PE | 4.062946301 | 4.37257E-06 |
| GM3(d37:5)-H | GM3 | 4.056795484 | 0.002161969 |
| Hex1Cer(d40:1)+HCOO | Hex1Cer | 4.056724795 | 0.025219325 |
| PI(18:1e_18:1)-H | PI | 4.050112456 | 0.023086342 |
| GM2(d32:1)-H | GM2 | 4.049190388 | 0.028257261 |
| Cer(d18:1_24:1)+HCOO | Cer | 4.047294405 | 0.004010781 |
| PE(18:1p_22:1)+H | PE | 4.041754235 | 0.023882111 |
| CL(66:3)-2H | CL | 4.030252724 | 0.011305837 |
| PE(16:1_16:1)-H | PE | 4.027798033 | 0.011140768 |
| LPE(18:1)-H | LPE | 4.025548569 | 8.38597E-06 |
| PS(17:0_18:1)-H | PS | 4.015454864 | 0.007005386 |
| DG(30:0e)+Na | DG | 4.012645855 | 0.000156177 |
| SPH(d17:1)+H | SPH | 4.004351302 | 0.000239537 |
| PC(15:0_18:1)+H | PC | 4.003045293 | 4.90834E-07 |
| Cer(m19:1_23:3)+H | Cer | 3.985892614 | 1.24252E-05 |
| StE(24:7)+H | StE | 3.985322161 | 2.61537E-06 |
| PG(16:0_16:1)+NH4 | PG | 3.983828829 | 0.010151288 |
| Hex1Cer(d46:2)+HCOO | Hex1Cer | 3.971746561 | 0.008916384 |
| PA(20:0e_22:6)-H | PA | 3.970361987 | 0.012168817 |
| PC(19:1_18:1)+H | PC | 3.962722479 | 0.001144632 |
| Hex1Cer(m19:0_23:3)+H | Hex1Cer | 3.962569494 | 0.005505011 |
| Cer(d18:1_26:1)+HCOO | Cer | 3.961476241 | 0.00630421 |
| PC(39:6)+H | PC | 3.961224638 | 0.00283055 |
| PI(19:1_18:1)-H | PI | 3.954955763 | 0.034864406 |
| Cer(d18:1_24:1)+H | Cer | 3.948358987 | 0.005244313 |
| TG(16:0e_16:0_16:1)+NH4 | TG | 3.942575414 | 4.82917E-05 |
| LPC(30:1)+Na | LPC | 3.936889179 | 0.003901804 |
| PE(18:1_18:2)+Na | PE | 3.923787364 | 0.000346958 |
| LPE(18:1p)+H | LPE | 3.920996054 | 0.000461671 |
| GM2(d36:1)-H | GM2 | 3.910801344 | 0.047778848 |
| Hex1Cer(t34:0)+HCOO | Hex1Cer | 3.90594347 | 0.008425249 |
| PC(18:1_14:1)+HCOO | PC | 3.898997088 | 0.012358109 |
| PC(29:1)+H | PC | 3.887551753 | 1.02501E-05 |
| Cer(d40:1)+HCOO | Cer | 3.887087221 | 0.00916377 |
| LPE(22:6)-H | LPE | 3.886036579 | 2.08673E-05 |
| Hex1Cer(d18:0_22:0)-H | Hex1Cer | 3.883177048 | 0.011355713 |
| PC(35:2e)+H | PC | 3.876498896 | 0.013739385 |
| Hex1Cer(d18:1_14:0)-H | Hex1Cer | 3.87422584 | 0.019852515 |
| PE(18:2e_22:1)-H | PE | 3.864265958 | 0.006081741 |
| Hex3Cer(d34:1)+HCOO | Hex3Cer | 3.85621245 | 0.041860308 |
| Hex2Cer(d18:1_16:0)+H | Hex2Cer | 3.85415104 | 0.008126595 |
| PE(18:1p_22:4)+H | PE | 3.853080005 | 5.81554E-05 |
| PG(18:1_18:1)-H | PG | 3.849653513 | 0.016416925 |
| Hex1Cer(m40:0+O)+HCOO | Hex1Cer | 3.83980404 | 0.010117963 |
| PI(18:1_20:4)-H | PI | 3.837269105 | 0.049567588 |
| Hex1Cer(m19:0_23:2)+H | Hex1Cer | 3.836202025 | 0.00206419 |
| DG(18:0_18:1)+NH4 | DG | 3.820960485 | 0.046929702 |
| Cer(d18:1_22:0)+HCOO | Cer | 3.815142407 | 0.001331988 |
| PE(16:0p_16:0)+H | PE | 3.804753441 | 1.54154E-05 |
| DG(34:0e)+Na | DG | 3.801745116 | 3.12233E-05 |
| SM(d38:2)+HCOO | SM | 3.79901348 | 0.011111226 |
| PE(16:0p_22:1)+H | PE | 3.783589405 | 1.54336E-05 |
| PC(16:2e_19:0)+H | PC | 3.782342121 | 1.65779E-05 |
| PC(16:0e_12:0)+HCOO | PC | 3.778768876 | 0.044431511 |
| TG(18:1_12:3_14:4)+Na | TG | 3.778223495 | 4.71938E-05 |
| TG(12:1e_10:4_22:6)+Na | TG | 3.771498073 | 0.006253452 |
| LPC(24:1)+H | LPC | 3.760190586 | 0.014965708 |
| PE(16:0_16:0)-H | PE | 3.760050605 | 0.000126666 |
| PC(16:1e_16:1)+HCOO | PC | 3.759787324 | 0.00507154 |
| PC(18:2_18:2)-CH3 | PC | 3.75663025 | 0.001775684 |
| LPE(20:4)-H | LPE | 3.741813203 | 3.87263E-06 |
| GM3(d32:1)-H | GM3 | 3.73794206 | 0.012653262 |
| PE(16:0p_20:0)+H | PE | 3.731308881 | 0.000486213 |
| DG(36:2e)+Na | DG | 3.731106693 | 0.033415353 |
| CL(78:9)-2H | CL | 3.728490678 | 0.003660801 |
| PE(18:1p_20:2)+Na | PE | 3.72822893 | 0.001576897 |
| Hex1Cer(m33:0)+HCOO | Hex1Cer | 3.725367846 | 0.004569921 |
| PS(16:1_14:0)-H | PS | 3.721497163 | 0.000372176 |
| PC(37:4)+H | PC | 3.720637228 | 0.003302862 |
| PE(18:1p_20:4)+H | PE | 3.716321662 | 3.87552E-06 |
| GM2(d40:2)-H | GM2 | 3.712840112 | 0.026227903 |
| PC(40:4e)+H | PC | 3.710585616 | 0.028941945 |
| CL(70:3)-2H | CL | 3.710166131 | 0.001988289 |
| PE(10:0_11:2)-H | PE | 3.709586905 | 0.007916605 |
| SM(d38:4)+HCOO | SM | 3.704504959 | 0.001020651 |
| Hex1Cer(d18:1_22:0)-H | Hex1Cer | 3.69498399 | 0.007144252 |
| CerP(d27:1)+H | CerP | 3.693848647 | 0.000457444 |
| PC(35:0e)+H | PC | 3.686503835 | 4.02145E-05 |
| LPE(18:1)+H | LPE | 3.675586939 | 0.00672474 |
| PE(39:2e)+H | PE | 3.66289697 | 0.000976183 |
| PE(18:0p_20:0)+H | PE | 3.660874968 | 4.81538E-05 |
| PC(16:1_14:0)+HCOO | PC | 3.659130472 | 0.004709364 |
| PC(16:0_16:0)-CH3 | PC | 3.657084323 | 0.000584158 |
| PE(18:0_16:0)-H | PE | 3.656298824 | 0.000580526 |
| PE(18:1_22:0)-H | PE | 3.645963637 | 0.010462647 |
| Cer(d18:0_22:0)+H | Cer | 3.639121258 | 0.006609684 |
| PC(40:8e)+H | PC | 3.638416967 | 0.010472225 |
| PS(40:3e)-H | PS | 3.637812814 | 0.009497705 |
| PE(16:1e_22:1)+H | PE | 3.635540067 | 0.045178505 |
| WE(19:1_17:3)+NH4 | WE | 3.631406227 | 0.019105737 |
| LPE(16:1e)-H | LPE | 3.630765938 | 7.67748E-06 |
| SM(d18:1_24:2)+H | SM | 3.624416387 | 2.51018E-05 |
| GM3(m33:0)+HCOO | GM3 | 3.620701812 | 0.022148224 |
| Hex2Cer(d32:2)-H | Hex2Cer | 3.61299024 | 0.029489379 |
| Cer(d18:1_14:0)+HCOO | Cer | 3.606173392 | 0.001255123 |
| PE(18:1p_22:3)+H | PE | 3.605749005 | 1.00062E-05 |
| PC(18:1_21:1)+H | PC | 3.60297416 | 0.000193113 |
| PE(18:0_20:1)-H | PE | 3.601031585 | 0.005999271 |
| GM3(d36:1)-H | GM3 | 3.598292132 | 0.034514962 |
| Cer(m42:1)+HCOO | Cer | 3.595588611 | 5.7102E-07 |
| PI(18:1_18:2)-H | PI | 3.588386046 | 0.037185855 |
| Hex1Cer(d38:0)+HCOO | Hex1Cer | 3.585001959 | 0.0192125 |
| PC(35:1e)+H | PC | 3.584361288 | 0.001500566 |
| GM3(d34:1)-H | GM3 | 3.57531068 | 0.016102466 |
| PE(18:0p_22:1)+H | PE | 3.571157312 | 1.15191E-05 |
| SPH(d17:0)+H | SPH | 3.569972172 | 9.54066E-05 |
| PC(11:0_16:0)+H | PC | 3.569298394 | 0.007454711 |
| Cer(m18:0_24:0)+H | Cer | 3.566432428 | 0.000645709 |
| Cer(d18:1_16:0)+H | Cer | 3.565973823 | 0.017740664 |
| PE(18:1_22:2)-H | PE | 3.562696561 | 0.003103195 |
| PC(18:1e_24:1)+HCOO | PC | 3.561534861 | 0.002533524 |
| PC(18:1e_20:4)+HCOO | PC | 3.560335066 | 0.002299034 |
| PI(18:0e_18:1)-H | PI | 3.559796928 | 0.006552516 |
| LPE(24:0)-H | LPE | 3.552384931 | 0.001527401 |
| Hex2Cer(d34:1)-H | Hex2Cer | 3.540152355 | 0.008395281 |
| phSM(d18:1_21:2)+HCOO | phSM | 3.539792372 | 0.003221985 |
| Hex1Cer(m38:0+O)+HCOO | Hex1Cer | 3.529565033 | 0.018617975 |
| TG(16:0e_16:0_18:1)+NH4 | TG | 3.529548937 | 0.000427422 |
| PE(42:3e)+Na | PE | 3.525052337 | 3.30761E-05 |
| PE(18:1p_18:1)+H | PE | 3.52097609 | 0.006358241 |
| PE(16:0p_14:1)+H | PE | 3.514833435 | 0.001135096 |
| WE(14:0_17:3)+NH4 | WE | 3.506811164 | 0.004281491 |
| PE(18:0p_6:0)+H | PE | 3.503191365 | 0.000151222 |
| PC(38:3e)+H | PC | 3.502379515 | 0.002465403 |
| PE(16:1_18:1)-H | PE | 3.481712738 | 0.010160434 |
| SM(d44:4)+H | SM | 3.48134775 | 0.012545386 |
| PE(18:1_22:1)-H | PE | 3.478067168 | 0.00578234 |
| PC(16:0_14:2)+H | PC | 3.475459827 | 0.002897093 |
| PE(18:1_14:0)-H | PE | 3.473824321 | 0.009823951 |
| LPC(20:0e)+H | LPC | 3.463484889 | 0.000278021 |
| TG(20:3e_18:1_18:1)+H | TG | 3.45966567 | 0.002889537 |
| Hex3Cer(d40:1)+HCOO | Hex3Cer | 3.45954351 | 0.037365821 |
| PS(42:5)-H | PS | 3.454430697 | 0.006268073 |
| PE(18:1p_20:5)+H | PE | 3.449309771 | 3.74152E-06 |
| Hex1Cer(m19:0_25:3)+H | Hex1Cer | 3.446038995 | 0.002086323 |
| PE(18:1e_22:2)+Na | PE | 3.444987977 | 0.000233477 |
| PE(16:0p_6:0)+H | PE | 3.438154867 | 0.002815449 |
| TG(42:10)+H | TG | 3.436783698 | 0.000127008 |
| PC(18:2e_18:1)+HCOO | PC | 3.436081986 | 0.018196258 |
| Hex1Cer(d18:1_16:0)+H | Hex1Cer | 3.431537291 | 0.009218281 |
| PC(38:1e)-CH3 | PC | 3.430727424 | 0.002866478 |
| PE(12:0p_6:0)+H | PE | 3.430617886 | 0.001092769 |
| Cer(m19:0_25:3)+H | Cer | 3.426615017 | 0.001058208 |
| PC(18:1_18:2)+HCOO | PC | 3.423652005 | 0.049854485 |
| TG(48:12e)+Na | TG | 3.422338924 | 0.000558825 |
| TG(16:0e_16:0_20:4)+H | TG | 3.421809523 | 0.001518681 |
| PE(38:0e)-H | PE | 3.41196779 | 0.037729844 |
| PS(18:0_22:3)+H | PS | 3.404712284 | 0.006046306 |
| Hex2Cer(d34:0)+HCOO | Hex2Cer | 3.392330574 | 0.018227233 |
| Cer(m20:1_18:1)+H | Cer | 3.391196425 | 0.001216532 |
| PS(42:4e)-H | PS | 3.390840684 | 0.011682121 |
| PE(18:1p_24:1)+H | PE | 3.389018488 | 3.68661E-05 |
| PE(18:2e_18:1)-H | PE | 3.387929875 | 0.009459838 |
| PE(18:1e_22:4)-H | PE | 3.38754461 | 0.017490954 |
| PE(16:1e_22:1)-H | PE | 3.3830398 | 0.006725744 |
| PE(19:0_18:1)-H | PE | 3.38218826 | 0.004256773 |
| PE(18:1e_16:0)-H | PE | 3.37401261 | 0.005146896 |
| PC(37:1)+H | PC | 3.371575278 | 0.001011125 |
| PC(17:0_18:1)-CH3 | PC | 3.369760336 | 0.004035564 |
| PC(36:3e)+HCOO | PC | 3.365937627 | 0.005514378 |
| PE(16:1e_19:0)+Na | PE | 3.36363712 | 0.004851587 |
| LPE(24:1)-H | LPE | 3.359367962 | 0.000583227 |
| PE(18:1e_20:0)-H | PE | 3.359190519 | 0.005777591 |
| TG(18:0e_16:0_18:1)+NH4 | TG | 3.356241023 | 0.000300444 |
| Hex2Cer(m34:0+O)+HCOO | Hex2Cer | 3.355885282 | 0.022641861 |
| PC(16:0e_16:1)+HCOO | PC | 3.355815128 | 0.002383605 |
| PE(16:0p_20:1)+H | PE | 3.348631325 | 3.23156E-05 |
| TG(42:9e)+Na | TG | 3.347654655 | 0.003030075 |
| SM(d42:2)+HCOO | SM | 3.340081255 | 0.003961382 |
| Hex1Cer(d38:2)+HCOO | Hex1Cer | 3.337414044 | 0.002415414 |
| SPH(d19:0)+H | SPH | 3.335816944 | 8.15538E-07 |
| Cer(d18:0_16:0)+H | Cer | 3.32860809 | 0.00412067 |
| PC(18:1_14:1)+H | PC | 3.326263982 | 3.67399E-05 |
| PC(42:6)+H | PC | 3.326239331 | 0.00138137 |
| PS(38:3e)-H | PS | 3.325983652 | 0.006356523 |
| TG(18:0e_18:1_18:1)+NH4 | TG | 3.324469095 | 0.000124717 |
| Cer(d18:0_14:0)+H | Cer | 3.323429005 | 0.009812029 |
| PC(52:5)-CH3 | PC | 3.321802643 | 0.040202542 |
| SPH(t17:0)+H | SPH | 3.321217336 | 0.00358101 |
| PS(16:0_14:0)-H | PS | 3.311244813 | 0.000161719 |
| DG(18:1_18:1)+NH4 | DG | 3.310214569 | 0.037106123 |
| DG(38:3e)+H | DG | 3.308537575 | 0.011425917 |
| LPE(22:1)-H | LPE | 3.304307555 | 1.22981E-06 |
| PC(32:3)+H | PC | 3.301983268 | 0.019875561 |
| PE(18:1e_22:1)-H | PE | 3.299787183 | 0.005069347 |
| PC(50:5)-CH3 | PC | 3.291920668 | 0.022359177 |
| SM(d40:2)+HCOO | SM | 3.291584661 | 0.006848045 |
| GM2(d38:1)-H | GM2 | 3.291297985 | 0.023564962 |
| DG(18:0_22:5)+NH4 | DG | 3.283947655 | 0.015416878 |
| PE(18:2e_24:1)-H | PE | 3.283772138 | 0.020669831 |
| TG(28:0_10:3_11:1)+Na | TG | 3.282892525 | 0.000535114 |
| TG(18:0e_16:0_18:0)+NH4 | TG | 3.280319603 | 0.001727378 |
| PI(18:0_22:6)-H | PI | 3.278292833 | 0.019829346 |
| PC(18:1e_18:1)+HCOO | PC | 3.277123013 | 0.029692167 |
| Cer(d36:0)+HCOO | Cer | 3.275023607 | 0.000561481 |
| TG(48:11e)+Na | TG | 3.265709471 | 0.034231303 |
| PE(18:2e_22:6)-H | PE | 3.262314099 | 0.007327831 |
| PC(37:3)+H | PC | 3.261231863 | 0.000146268 |
| SM(d44:2)+HCOO | SM | 3.26060216 | 0.001100826 |
| PC(16:0_16:1)+HCOO | PC | 3.257179673 | 0.001388541 |
| PC(18:1_22:2)-CH3 | PC | 3.251582476 | 0.020034543 |
| TG(18:0e_16:0_20:4)+H | TG | 3.249628917 | 0.000475259 |
| PC(30:1e)+H | PC | 3.242762509 | 0.003987342 |
| CL(66:1)-2H | CL | 3.237072986 | 0.01014717 |
| PE(18:0_20:4)-H | PE | 3.23671652 | 0.016373669 |
| Hex2Cer(d42:3)+HCOO | Hex2Cer | 3.235549074 | 0.003145338 |
| Hex1Cer(d30:1)+HCOO | Hex1Cer | 3.232907395 | 0.003002795 |
| CL(75:4)+H | CL | 3.232653614 | 0.007940095 |
| phSM(t39:2)+H | phSM | 3.22955395 | 0.003687206 |
| TG(6:0_14:2_20:5)+Na | TG | 3.225965799 | 6.6799E-05 |
| SPHP(d18:0)+H-H2O | SPHP | 3.225827848 | 0.001720742 |
| GM3(d40:2)-H | GM3 | 3.225182701 | 0.040591112 |
| TG(18:1e_16:0_18:4)+H | TG | 3.222163257 | 0.006980304 |
| PE(16:0e)+H | PE | 3.219594337 | 8.97157E-05 |
| PC(33:2)+H | PC | 3.218227455 | 0.049184805 |
| Cer(m32:0+O)+HCOO | Cer | 3.215536558 | 0.000463403 |
| PE(20:2e_24:1)+H | PE | 3.213807927 | 0.000771015 |
| PI(18:1_18:1)-H | PI | 3.204152453 | 0.04180627 |
| PC(20:1_18:1)+HCOO | PC | 3.20413926 | 0.004957619 |
| Cer(d18:1_20:0)+HCOO | Cer | 3.195078216 | 0.001668476 |
| DG(36:3e)+Na | DG | 3.188351518 | 0.012623589 |
| SQDG(36:7)+HCOO | SQDG | 3.184706901 | 0.002635631 |
| SM(d38:0)+H | SM | 3.182505407 | 0.015661626 |
| PE(8:0e_10:1)+Na | PE | 3.178428253 | 0.000299217 |
| PE(18:1e)+H | PE | 3.176139173 | 8.86469E-05 |
| CL(70:5)-2H | CL | 3.16756862 | 0.000512722 |
| PC(18:1_22:1)+HCOO | PC | 3.167286622 | 0.006409606 |
| TG(18:1e_16:0_18:1)+NH4 | TG | 3.157866389 | 0.000246173 |
| PE(8:1e_10:0)+Na | PE | 3.15190062 | 0.00083984 |
| Cer(d18:1_24:0)+HCOO | Cer | 3.147257302 | 0.007635552 |
| PC(33:2)+H | PC | 3.145279385 | 6.58229E-05 |
| PE(16:0_18:3)-H | PE | 3.136557198 | 0.000187589 |
| PE(18:1p_21:1)+H | PE | 3.133865847 | 0.002379266 |
| PC(35:1e)+H | PC | 3.133267004 | 0.007550976 |
| GM3(d32:0)+H | GM3 | 3.132802402 | 6.91364E-05 |
| phSM(t34:1)-CH3 | phSM | 3.132015718 | 0.003671309 |
| PE(18:1_24:1)-H | PE | 3.13145768 | 0.006402964 |
| PI(18:1e_16:0)-H | PI | 3.128448876 | 0.038332051 |
| Cer(m18:1_16:0)+H | Cer | 3.128205765 | 0.001138205 |
| PE(16:0p_18:1)+H | PE | 3.126206309 | 8.70459E-07 |
| PC(40:6)+H | PC | 3.1119865 | 0.003375752 |
| GM3(m32:0+O)-H | GM3 | 3.109392662 | 0.01653397 |
| SM(d32:1)+HCOO | SM | 3.10913954 | 0.003275951 |
| LPE(22:4)-H | LPE | 3.107882729 | 0.0001439 |
| DG(38:4e)+H | DG | 3.104196166 | 0.042109961 |
| PC(16:0e_20:1)+HCOO | PC | 3.099604815 | 0.008466489 |
| PS(18:0_20:3)+H | PS | 3.097787092 | 0.00085797 |
| PC(15:0_14:0)+HCOO | PC | 3.097739433 | 0.014556188 |
| Cer(d12:0_20:0)+HCOO | Cer | 3.095059048 | 0.000138397 |
| Hex1Cer(d18:1_22:1)+HCOO | Hex1Cer | 3.093788715 | 0.008979014 |
| LPC(17:1)+H | LPC | 3.09093675 | 0.00093012 |
| Cer(m19:0_23:2)+H | Cer | 3.089402427 | 1.38703E-05 |
| PE(18:1p_19:1)+H | PE | 3.088206796 | 0.005266636 |
| Cer(d18:1_16:0)+H | Cer | 3.087348818 | 0.002162577 |
| Cer(d42:2)+HCOO | Cer | 3.085836933 | 0.035005385 |
| TG(18:0e_16:1_16:1)+NH4 | TG | 3.081302497 | 0.000363024 |
| TG(16:0e_16:0_22:1)+NH4 | TG | 3.073470523 | 0.001325755 |
| PC(37:6)+H | PC | 3.073024787 | 0.00016825 |
| PG(16:0_18:1)-H | PG | 3.065393764 | 0.018684646 |
| PS(42:5e)-H | PS | 3.065135531 | 0.008462779 |
| PC(14:1e_18:1)+H | PC | 3.059445726 | 0.037162391 |
| PE(18:1p_24:2)+H | PE | 3.059403 | 0.000395355 |
| PC(52:4)-CH3 | PC | 3.058905496 | 0.011460472 |
| LPE(18:1e)-H | LPE | 3.057040186 | 2.77813E-06 |
| Cer(m18:0_18:0)+H | Cer | 3.055143217 | 0.009354971 |
| PS(42:3)-H | PS | 3.047869867 | 0.009707713 |
| PC(16:1_14:1)+H | PC | 3.047411992 | 0.005595506 |
| LPE(22:5)-H | LPE | 3.046725928 | 3.34527E-05 |
| CL(73:1)-H | CL | 3.04411342 | 0.042188955 |
| PC(30:2)+H | PC | 3.04107324 | 0.02557604 |
| PE(18:2e_14:1)-H | PE | 3.034868938 | 0.001292894 |
| Hex1Cer(d44:1)+HCOO | Hex1Cer | 3.017513494 | 0.023448103 |
| SQDG(38:8)+HCOO | SQDG | 3.01301052 | 0.024940561 |
| PC(31:0e)+H | PC | 3.010512747 | 0.044218821 |
| PC(18:0e_16:0)+HCOO | PC | 3.009945154 | 0.01304286 |
| PE(26:1_18:1)-H | PE | 3.009213648 | 0.02393713 |
| PS(18:1_22:0)-H | PS | 2.995355562 | 0.022056604 |
| PE(18:0_18:1)-H | PE | 2.988864425 | 0.005931084 |
| PC(18:1e_24:2)+HCOO | PC | 2.982951983 | 0.049740698 |
| PS(37:3)-H | PS | 2.982914139 | 0.002283516 |
| PE(20:1_18:1)-H | PE | 2.981944435 | 0.005622541 |
| Cer(d18:0_24:1)+H | Cer | 2.977476501 | 0.003846107 |
| TG(16:0e_18:1_20:4)+H | TG | 2.975813468 | 0.001787598 |
| PS(16:0e_16:1)-H | PS | 2.974423146 | 0.03312854 |
| PC(38:0e)+H | PC | 2.97308802 | 0.007723268 |
| Cer(d18:1_16:0)+HCOO | Cer | 2.965695745 | 0.000213378 |
| Hex1Cer(d42:1)-H | Hex1Cer | 2.961904686 | 0.013686416 |
| PC(33:0e)+H | PC | 2.961010951 | 0.001906224 |
| PI(18:0_18:1)-H | PI | 2.959525823 | 0.018067025 |
| PE(16:0_20:5)-H | PE | 2.958650294 | 0.015426389 |
| SM(t34:1)+H | SM | 2.957285141 | 1.48138E-05 |
| Hex1Cer(d18:1_26:1)+HCOO | Hex1Cer | 2.951831947 | 0.012947416 |
| CL(74:9)-2H | CL | 2.93827548 | 0.018905725 |
| PE(18:0e_16:0)-H | PE | 2.937839313 | 0.000670711 |
| PE(18:1_24:2)-H | PE | 2.936660261 | 0.009084351 |
| Hex1Cer(d18:1_24:0)-H | Hex1Cer | 2.935912678 | 0.016181175 |
| PE(20:2e)+Na | PE | 2.935059356 | 1.82977E-05 |
| PE(36:0e)-H | PE | 2.932221155 | 0.00529558 |
| Cer(t16:0_18:0)+HCOO | Cer | 2.929044696 | 0.00167427 |
| PE(18:0p_24:1)+H | PE | 2.929005593 | 0.002381295 |
| PE(18:0p_20:4)+H | PE | 2.928535536 | 0.000158028 |
| Hex1Cer(m19:0_25:2)+H | Hex1Cer | 2.927451295 | 0.009935571 |
| PI(18:0_18:1)+Na | PI | 2.921285121 | 0.008321001 |
| PS(12:1e_6:0)+H | PS | 2.919358427 | 0.001466193 |
| PC(18:1_24:1)+HCOO | PC | 2.916534431 | 0.016901092 |
| PS(16:1_16:1)-H | PS | 2.915884911 | 0.000224481 |
| PC(26:1_18:1)+HCOO | PC | 2.91062329 | 0.027938053 |
| Cer(d24:2_17:0)+HCOO | Cer | 2.910608696 | 0.005500075 |
| LPC(18:0)+HCOO | LPC | 2.900049152 | 0.000351381 |
| Hex1Cer(d18:1_24:1)-H | Hex1Cer | 2.898188976 | 0.012363501 |
| GM3(d40:1)-H | GM3 | 2.896774781 | 0.031097802 |
| PC(50:2)-CH3 | PC | 2.883076766 | 0.007110259 |
| PC(18:1_18:1)+HCOO | PC | 2.878901689 | 0.000230509 |
| PE(18:0e_18:1)-H | PE | 2.8727262 | 0.011059047 |
| CL(78:3)-2H | CL | 2.872522593 | 0.005205483 |
| LPS(37:1)-H | LPS | 2.86895656 | 0.014117192 |
| PC(36:4)+H | PC | 2.867186854 | 0.010648002 |
| PA(44:6)-H | PA | 2.866503332 | 0.004039108 |
| TG(44:10e)+Na | TG | 2.865724679 | 0.024309966 |
| SM(d35:2)+H | SM | 2.865114212 | 0.000240057 |
| PS(18:0_20:5)-H | PS | 2.861633103 | 0.04521712 |
| Hex1Cer(d34:2+2O)+HCOO | Hex1Cer | 2.860235807 | 0.001078505 |
| PE(37:3e)+Na | PE | 2.85713801 | 0.024729112 |
| PI(18:0_20:1)-H | PI | 2.852898767 | 0.024751192 |
| Cer(d43:2)+HCOO | Cer | 2.850705416 | 0.030306167 |
| PE(16:1e_16:0)-H | PE | 2.850143067 | 0.000188689 |
| PE(50:2)+H | PE | 2.84809627 | 9.01061E-05 |
| PE(16:0p_20:5)+H | PE | 2.8431741 | 2.64315E-06 |
| GM2(d40:1)-H | GM2 | 2.841807074 | 0.030880478 |
| PC(52:3)-CH3 | PC | 2.841310541 | 0.023525514 |
| CerP(d39:0+O)+NH4 | CerP | 2.832046633 | 0.010610669 |
| Cer(t18:0_24:0)+H-H2O | Cer | 2.827926146 | 0.011601269 |
| PC(16:0e_24:1)+HCOO | PC | 2.827679649 | 0.025669208 |
| Cer(m19:0_23:3)+H | Cer | 2.824552431 | 0.022682937 |
| CerP(d14:0_15:1)+H | CerP | 2.822648113 | 7.20334E-05 |
| PE(20:5e)+H | PE | 2.820997518 | 0.002279148 |
| PE(18:1p_14:1)+H | PE | 2.820530241 | 0.035302281 |
| PA(18:0e_16:0)-H | PA | 2.819166281 | 0.010109474 |
| Cer(d34:0+O)+HCOO | Cer | 2.818758934 | 0.000761127 |
| PE(16:0p_19:1)+H | PE | 2.81308599 | 0.00042249 |
| LPE(20:5)-H | LPE | 2.810572014 | 8.99458E-05 |
| PE(18:0e_6:0)+H | PE | 2.806313238 | 0.000992321 |
| Hex1Cer(m19:0_23:2)+H | Hex1Cer | 2.805160418 | 0.027100873 |
| SM(d33:0)+H | SM | 2.795516037 | 0.009684998 |
| PC(48:2)-CH3 | PC | 2.794458019 | 0.030524023 |
| PC(18:2e_20:4)+HCOO | PC | 2.793264743 | 0.043506563 |
| PE(52:2)+Na | PE | 2.790191433 | 0.036831991 |
| Hex1Cer(d42:0)+HCOO | Hex1Cer | 2.787805969 | 0.020778316 |
| TG(16:0e_18:1_22:2)+NH4 | TG | 2.781314796 | 0.003497423 |
| PC(18:1_21:0)+H | PC | 2.779117347 | 1.87602E-05 |
| Hex3Cer(d42:1)+HCOO | Hex3Cer | 2.77834653 | 0.013903019 |
| PS(16:1_18:1)-H | PS | 2.777007654 | 0.006085067 |
| CL(68:3)-2H | CL | 2.776170392 | 0.041924727 |
| PE(18:1e_22:6)-H | PE | 2.76740451 | 0.001663191 |
| Hex2Cer(m38:3)-H | Hex2Cer | 2.765267863 | 0.013561814 |
| PS(16:0_16:1)-H | PS | 2.762682014 | 0.037332364 |
| PC(50:2)-CH3 | PC | 2.75808718 | 0.014791046 |
| PE(18:2e_19:1)-H | PE | 2.757048048 | 0.013683706 |
| TG(16:0e_18:1_20:2)+NH4 | TG | 2.756216777 | 0.007102451 |
| GM3(m42:3)+H | GM3 | 2.749068653 | 0.022635368 |
| SPH(m17:0)+H | SPH | 2.748482928 | 0.001535334 |
| Hex2Cer(d18:0_16:0)+H | Hex2Cer | 2.744923721 | 0.028261509 |
| GM2(d33:1)-H | GM2 | 2.732253068 | 0.047498396 |
| PE(42:7e)-H | PE | 2.728337942 | 0.002549712 |
| Cer(t35:0+O)+H | Cer | 2.72706596 | 0.00210061 |
| LPE(18:3)-H | LPE | 2.719458809 | 0.000738327 |
| PC(18:2e_18:2)+HCOO | PC | 2.716611853 | 0.001219375 |
| PC(37:5)+H | PC | 2.708742291 | 2.31963E-05 |
| CL(87:9)-2H | CL | 2.707328992 | 0.020430977 |
| CL(79:7)-2H | CL | 2.705800988 | 0.003644537 |
| PE(50:2)+Na | PE | 2.696805704 | 0.003626157 |
| PE(34:1_18:2)-H | PE | 2.696110377 | 0.028556755 |
| PE(18:1_18:1)-H | PE | 2.695981283 | 0.014727008 |
| PE(16:1e_16:1)-H | PE | 2.693967241 | 0.003576641 |
| PE(18:3e_22:2)+H | PE | 2.690079152 | 0.001984016 |
| GM3(d40:0)+H | GM3 | 2.685566273 | 0.010338669 |
| PI(18:0_22:5)-H | PI | 2.683357423 | 0.041780359 |
| PC(40:7e)-CH3 | PC | 2.681219277 | 0.015574517 |
| PC(16:0_20:1)+HCOO | PC | 2.681138958 | 0.027527143 |
| Cer(d33:0)+H | Cer | 2.681042041 | 7.04613E-05 |
| LPS(37:0)+H | LPS | 2.677593886 | 0.049342071 |
| GD1a(d34:1)-2H | GD1a | 2.676613501 | 0.004698161 |
| PS(40:4e)-H | PS | 2.676090926 | 0.014206734 |
| SM(d42:2)+H | SM | 2.670691917 | 0.001196979 |
| Cer(t17:0_25:1)+HCOO | Cer | 2.666997466 | 0.017042963 |
| PE(18:1e_22:3)+Na | PE | 2.664430402 | 0.004563082 |
| PS(20:0_18:1)-H | PS | 2.664221523 | 0.005337646 |
| PS(18:1_22:1)-H | PS | 2.663272262 | 0.023767616 |
| PE(37:1e)+H | PE | 2.662129765 | 0.002997451 |
| PC(16:1_18:1)+HCOO | PC | 2.655114687 | 0.013503685 |
| CL(70:1)-2H | CL | 2.652899618 | 0.001515328 |
| CL(78:7)-2H | CL | 2.648813981 | 0.018835489 |
| PS(19:1_18:0)-H | PS | 2.645886512 | 0.000166111 |
| PC(16:0_24:1)+HCOO | PC | 2.643320903 | 0.023631815 |
| SM(d32:2)+HCOO | SM | 2.639352725 | 0.004515655 |
| Cer(d18:0_24:0)+H | Cer | 2.638943277 | 2.44053E-05 |
| ZyE(24:7)+H | ZyE | 2.637124327 | 5.83051E-05 |
| Hex1Cer(d18:1_20:0)+HCOO | Hex1Cer | 2.628507592 | 0.004503718 |
| PI(17:0_18:1)-H | PI | 2.622291038 | 0.024913045 |
| Hex3Cer(d42:2)+HCOO | Hex3Cer | 2.61836937 | 0.036607737 |
| PC(52:2)-CH3 | PC | 2.614220416 | 0.029948529 |
| SM(d44:3)+HCOO | SM | 2.610615793 | 0.011858397 |
| Cer(d42:1+O)+HCOO | Cer | 2.609366438 | 0.019400655 |
| PE(16:0_18:1)-H | PE | 2.597613516 | 0.000968791 |
| PC(16:0e_18:1)+HCOO | PC | 2.592087765 | 0.005656368 |
| TG(16:0e_18:1_22:1)+NH4 | TG | 2.590719177 | 0.004927562 |
| GD1a(d42:2)-2H | GD1a | 2.587043726 | 0.001668443 |
| PE(18:1_24:0)-H | PE | 2.584222844 | 0.013144833 |
| PC(14:0_14:0)+HCOO | PC | 2.58274939 | 0.013448487 |
| PC(16:0_16:1)+HCOO | PC | 2.581179945 | 0.001385685 |
| PE(16:1e_20:1)-H | PE | 2.571630745 | 0.006898647 |
| CL(74:3)-H | CL | 2.569394188 | 0.007810842 |
| PE(16:0_18:1)-H | PE | 2.569099927 | 0.008265827 |
| PS(18:1_20:3)-H | PS | 2.566193779 | 0.019091959 |
| PS(40:6e)-H | PS | 2.563542344 | 0.002667813 |
| PE(18:2e_24:2)-H | PE | 2.559920257 | 0.012425055 |
| SM(d42:1)+HCOO | SM | 2.555821823 | 0.03423861 |
| PC(16:0_22:1)+HCOO | PC | 2.551411439 | 0.010210909 |
| CL(83:7)-2H | CL | 2.551058454 | 0.003046372 |
| PC(18:1_20:2)+HCOO | PC | 2.548672139 | 0.040575438 |
| PC(16:1e_18:1)+HCOO | PC | 2.543758839 | 0.004115389 |
| Hex2Cer(d18:1_24:1)+H | Hex2Cer | 2.542598724 | 0.00863866 |
| SM(d35:4)+H | SM | 2.53958297 | 0.03090657 |
| Hex1Cer(m19:0_23:3)+H | Hex1Cer | 2.538820293 | 0.026920553 |
| PS(18:1_18:1)+Na | PS | 2.538048387 | 0.001405045 |
| PS(20:1_18:1)-H | PS | 2.536233597 | 0.010595798 |
| PC(35:3)+H | PC | 2.534450272 | 0.013061715 |
| PE(52:2)+H | PE | 2.534137629 | 0.000310239 |
| CL(81:11)-2H | CL | 2.528500392 | 0.00521202 |
| LPS(18:1)-H | LPS | 2.526923459 | 0.000263304 |
| PC(16:1e_18:2)+HCOO | PC | 2.51780325 | 0.007507251 |
| PC(50:2)+H | PC | 2.512482807 | 1.28523E-05 |
| PC(12:0_14:0)+HCOO | PC | 2.509767249 | 0.001613461 |
| DG(18:0_20:4)+NH4 | DG | 2.501844032 | 0.044378705 |
| SM(d42:2)+HCOO | SM | 2.5011148 | 0.01006719 |
| PE(18:1e_20:4)-H | PE | 2.494297201 | 0.001160137 |
| PC(20:1_13:0)+H | PC | 2.488237274 | 0.029059233 |
| PC(34:1e)+H | PC | 2.488125418 | 0.025346047 |
| PS(18:0_22:3)-H | PS | 2.48699878 | 0.02991573 |
| PC(48:3)-CH3 | PC | 2.486705457 | 0.044720777 |
| Hex1Cer(d44:1)+HCOO | Hex1Cer | 2.481124376 | 0.040824784 |
| PE(16:1e_20:4)-H | PE | 2.471417288 | 0.037085958 |
| PC(50:3)-CH3 | PC | 2.467241017 | 0.026854755 |
| TG(30:1_11:3_11:3)+H | TG | 2.463794873 | 0.022917136 |
| Cer(d40:0)+HCOO | Cer | 2.461960468 | 0.008958002 |
| GM3(d33:1)-H | GM3 | 2.459748903 | 0.034641757 |
| ST(t38:1+O)+NH4 | ST | 2.459417768 | 0.015307593 |
| PE(17:0_18:1)-H | PE | 2.458595386 | 0.005196874 |
| PE(42:2e)-H | PE | 2.456641758 | 0.000533917 |
| GM3(d44:2)-H | GM3 | 2.456605987 | 0.047988607 |
| PE(16:1_18:2)-H | PE | 2.456412073 | 0.010533795 |
| PC(16:0_20:5)+HCOO | PC | 2.454466387 | 0.014106808 |
| PS(18:0_18:1)-H | PS | 2.447563731 | 0.035113845 |
| Cer(m40:0+O)+HCOO | Cer | 2.443752351 | 0.017025033 |
| SM(d30:1)+H | SM | 2.442960561 | 0.00041897 |
| PE(18:1_18:1)+H | PE | 2.436808785 | 0.002640353 |
| Hex1Cer(d33:1)+HCOO | Hex1Cer | 2.430544046 | 0.019327188 |
| PS(44:6e)-H | PS | 2.423392016 | 0.020527792 |
| PG(18:0_16:0)-H | PG | 2.422206451 | 0.003255921 |
| PE(16:0p_21:1)+H | PE | 2.418811921 | 0.002333386 |
| PC(33:2e)+H | PC | 2.417728459 | 0.016428681 |
| Cer(m18:0_22:0)+H | Cer | 2.410814585 | 0.006790453 |
| Hex1Cer(d18:1_18:0)+HCOO | Hex1Cer | 2.405821778 | 0.006008068 |
| PE(18:1e_20:4)-H | PE | 2.405041125 | 0.031744198 |
| PC(16:1_18:1)+HCOO | PC | 2.395775644 | 0.015914004 |
| PE(18:2e_22:5)-H | PE | 2.391831069 | 0.010201191 |
| CerP(d41:2)-H | CerP | 2.389851077 | 0.000125203 |
| PE(18:1p_22:5)+H | PE | 2.382980245 | 1.47506E-05 |
| PC(15:0_18:2)+HCOO | PC | 2.377433657 | 0.032543418 |
| DG(36:4e)+H | DG | 2.374716832 | 0.044012319 |
| PC(16:1e_24:1)-CH3 | PC | 2.374242386 | 0.000165559 |
| PS(38:6e)-H | PS | 2.369399166 | 0.000518883 |
| PC(18:0_16:0)+HCOO | PC | 2.367313562 | 0.010852319 |
| PC(26:1_16:0)+HCOO | PC | 2.36209102 | 0.029721288 |
| GM2(d42:2)-H | GM2 | 2.361371014 | 0.036812289 |
| LPS(18:0)-H | LPS | 2.360591461 | 0.000566068 |
| Cer(d18:0_24:1)+HCOO | Cer | 2.355997072 | 0.004786245 |
| PG(36:5)+Na | PG | 2.353872027 | 0.001474396 |
| Hex2Cer(t43:6)-H | Hex2Cer | 2.351786328 | 0.003624558 |
| SM(d32:0)+HCOO | SM | 2.349180817 | 0.019559461 |
| PC(15:0_16:1)+HCOO | PC | 2.34831041 | 0.024521323 |
| PC(16:0_18:1)+HCOO | PC | 2.346388388 | 0.022931545 |
| Cer(d18:0_16:0)+HCOO | Cer | 2.336924846 | 5.41084E-06 |
| Cer(m34:0+O)+HCOO | Cer | 2.334715264 | 4.06853E-06 |
| PC(19:0_18:1)+HCOO | PC | 2.326256232 | 0.031132265 |
| Hex1Cer(d42:1+O)+HCOO | Hex1Cer | 2.326026489 | 0.040694882 |
| PS(18:1_24:1)-H | PS | 2.321609246 | 0.017896994 |
| PE(18:2e_20:4)-H | PE | 2.317536335 | 0.007731838 |
| DG(34:2e)+Na | DG | 2.314262878 | 0.000158946 |
| phSM(t34:2)-CH3 | phSM | 2.305992866 | 0.004501899 |
| phSM(t36:1)-CH3 | phSM | 2.30457322 | 0.006192244 |
| PC(42:3e)+H | PC | 2.301911897 | 0.014636612 |
| PE(35:1e)-H | PE | 2.299824692 | 0.006853199 |
| SM(d36:1)+HCOO | SM | 2.299270981 | 0.007096938 |
| PS(42:7e)-H | PS | 2.297677122 | 0.009716476 |
| Hex1Cer(d42:2)+HCOO | Hex1Cer | 2.296789033 | 0.015849905 |
| LPS(20:1)-H | LPS | 2.293304708 | 0.00155056 |
| PS(18:4_18:1)-H | PS | 2.292239593 | 0.028834176 |
| PC(14:1e_17:0)-CH3 | PC | 2.284690597 | 0.009128224 |
| PC(16:0e_16:0)+HCOO | PC | 2.28216118 | 0.006659502 |
| PS(20:4e_22:2)-H | PS | 2.280073203 | 0.012050127 |
| Hex1Cer(t42:1)+HCOO | Hex1Cer | 2.274071956 | 0.034971197 |
| Cer(d44:1)+HCOO | Cer | 2.272728176 | 0.02347441 |
| CL(74:1)-2H | CL | 2.271982031 | 0.021296735 |
| Cer(m18:0_16:0)+H | Cer | 2.270665207 | 0.000118164 |
| SM(d43:5)+H | SM | 2.266463335 | 0.044259167 |
| SM(d14:0_22:5)+H | SM | 2.262166273 | 0.027732766 |
| PE(37:2e)+H | PE | 2.260742875 | 0.025906805 |
| PC(15:0_16:0)+Na | PC | 2.260074542 | 0.000107167 |
| PE(18:2e_21:1)-H | PE | 2.245396971 | 0.003216156 |
| PE(16:1e_18:1)-H | PE | 2.244042479 | 0.009811661 |
| PC(33:3)+H | PC | 2.240404775 | 3.39571E-05 |
| PC(18:0_22:5)+HCOO | PC | 2.238553526 | 0.005702398 |
| PS(16:0_16:0)-H | PS | 2.232921608 | 0.003072649 |
| PS(10:0e_6:0)+H | PS | 2.23014998 | 0.001714264 |
| SM(d42:1)+HCOO | SM | 2.2297358 | 0.024783452 |
| DG(36:2e)+Na | DG | 2.227311488 | 0.021628617 |
| PE(16:1e_17:0)-H | PE | 2.212801246 | 0.010094395 |
| DG(34:1e)+Na | DG | 2.208478078 | 0.03756036 |
| PC(28:1_16:0)+HCOO | PC | 2.20437305 | 0.034952793 |
| TG(16:0_14:0_16:0)+NH4 | TG | 2.182144028 | 0.037438927 |
| PS(16:0_18:1)-H | PS | 2.169416087 | 0.014787535 |
| PE(16:0p_17:1)+H | PE | 2.162662306 | 0.005284074 |
| PE(18:2e)+H | PE | 2.149901905 | 0.020336489 |
| SQDG(43:5)-H | SQDG | 2.146349689 | 0.016384106 |
| Cer(d34:1)+H | Cer | 2.144794081 | 0.013526214 |
| CL(72:1)-2H | CL | 2.143061016 | 0.047766967 |
| PA(22:6_21:0)-H | PA | 2.1429446 | 0.042224355 |
| LPE(18:2)-H | LPE | 2.140553287 | 2.49061E-05 |
| PC(15:0_16:0)+HCOO | PC | 2.137488399 | 0.00113639 |
| TG(16:0_16:0_16:0)+NH4 | TG | 2.134958851 | 0.016152607 |
| TG(16:0e_16:0_24:1)+NH4 | TG | 2.131638888 | 0.000290091 |
| Hex1Cer(d38:6)+HCOO | Hex1Cer | 2.130142059 | 0.029321888 |
| PS(12:0e_6:0)+H | PS | 2.128549552 | 0.007764554 |
| PE(16:1e_14:1)-H | PE | 2.127293743 | 0.003022037 |
| PS(17:1_18:1)-H | PS | 2.121822516 | 0.02425421 |
| Hex1Cer(t16:0_20:4)-H | Hex1Cer | 2.117276641 | 0.017478831 |
| SM(d36:0)+HCOO | SM | 2.115383398 | 0.005023424 |
| PG(20:0_18:1)-H | PG | 2.113947898 | 0.036420324 |
| DG(30:2e)+H | DG | 2.108032922 | 0.041733846 |
| CL(20:5_20:0_20:1_15:0)-2H | CL | 2.107423412 | 0.034669755 |
| PC(18:1_20:3)+HCOO | PC | 2.103982557 | 0.002978923 |
| DG(32:2e)+H | DG | 2.102704909 | 0.041056588 |
| MG(16:2e)+H | MG | 2.100614132 | 0.006199172 |
| PG(18:0_18:1)-H | PG | 2.089075434 | 0.024650655 |
| PG(35:3)+NH4 | PG | 2.088738803 | 0.001074684 |
| SM(d40:1)+HCOO | SM | 2.085574816 | 0.043386331 |
| PA(14:1e_11:4)-H | PA | 2.085196492 | 0.0038378 |
| PC(18:0_20:5)+HCOO | PC | 2.083234364 | 0.015619668 |
| TG(12:1e_23:0_23:0)+NH4 | TG | 2.077436705 | 0.002020355 |
| PC(16:0_16:0)+HCOO | PC | 2.068615894 | 0.032228656 |
| PC(16:0_20:4)+HCOO | PC | 2.057703936 | 0.01267252 |
| PC(18:0_20:3)+HCOO | PC | 2.049689944 | 0.047520718 |
| PC(18:1_22:6)+HCOO | PC | 2.049042294 | 0.025153204 |
| PC(16:0_22:5)+HCOO | PC | 2.047074108 | 0.031934952 |
| PS(36:5)-H | PS | 2.039420949 | 0.024512428 |
| PE(17:1_18:1)-H | PE | 2.037667974 | 0.012393798 |
| SM(d40:0)+HCOO | SM | 2.033815534 | 0.034430139 |
| TG(18:0_16:0_18:3)+H | TG | 2.024443342 | 0.005302792 |
| PC(16:0_17:0)+HCOO | PC | 2.024342004 | 0.013342756 |
| PC(18:0_22:6)+HCOO | PC | 2.020970126 | 0.019742128 |
| SQDG(36:4)-H | SQDG | 2.020309519 | 0.032726021 |
| PC(16:0_14:0)+HCOO | PC | 2.014204293 | 0.026844126 |
| PC(16:1_20:3)-CH3 | PC | 2.00077963 | 0.00757013 |
| SM(d34:2)+HCOO | SM | 1.999363947 | 0.014169898 |
| PE(18:1_20:3)-H | PE | 1.99096959 | 0.00753127 |
| PC(16:1_18:2)-CH3 | PC | 1.980898582 | 0.017004164 |
| PC(19:1_18:1)+HCOO | PC | 1.96323034 | 0.046721958 |
| LPS(16:0)-H | LPS | 1.960132832 | 0.000124931 |
| PC(18:1_18:2)+HCOO | PC | 1.9591896 | 0.030664153 |
| PG(37:4)+NH4 | PG | 1.957577474 | 0.024468077 |
| GD1a(m38:1)-2H | GD1a | 1.952617248 | 0.032427317 |
| Cer(m22:0_20:0+O)+HCOO | Cer | 1.952166258 | 0.003935468 |
| LPC(20:0)+H | LPC | 1.949315538 | 0.03763209 |
| PS(42:10e)-H | PS | 1.939972364 | 0.012345883 |
| PE(18:1e_20:5)-H | PE | 1.939826999 | 0.022795017 |
| DG(21:2e)+Na | DG | 1.939709566 | 0.007679823 |
| TG(18:0_16:0_16:0)+NH4 | TG | 1.93592432 | 0.006384375 |
| Hex3Cer(d18:0_16:0)+H | Hex3Cer | 1.935069188 | 8.26688E-05 |
| SM(d34:0)+HCOO | SM | 1.933968998 | 0.027911926 |
| SM(d36:2)+HCOO | SM | 1.932659939 | 0.043348558 |
| Hex1Cer(d18:0_16:0)-H | Hex1Cer | 1.93204303 | 0.006163815 |
| PE(26:0_18:1)-H | PE | 1.929696363 | 0.046211248 |
| PC(18:0_20:4)+HCOO | PC | 1.928175287 | 0.00766087 |
| Hex1Cer(d18:1_24:2)+HCOO | Hex1Cer | 1.928084314 | 0.006638148 |
| MG(28:0)+Na | MG | 1.916760519 | 0.027358776 |
| LPE(18:2)+H | LPE | 1.904672617 | 0.016839179 |
| Cer(m19:0_22:3)+H | Cer | 1.893542617 | 0.029369645 |
| PE(19:1_18:1)-H | PE | 1.882110963 | 0.025164453 |
| TG(16:0_14:0_14:0)+NH4 | TG | 1.878944475 | 0.03505952 |
| PC(17:1_18:1)+HCOO | PC | 1.868208097 | 0.040822802 |
| TG(18:3_12:3_14:4)+Na | TG | 1.86652719 | 0.03943985 |
| Cer(m18:1_24:0+O)+H | Cer | 1.863458125 | 0.024815034 |
| PS(18:0_22:5)-H | PS | 1.855655684 | 0.038395416 |
| GM3(d37:4+2O)-H | GM3 | 1.847368932 | 0.046967108 |
| TG(9:0_9:0_12:3)+H | TG | 1.844008872 | 0.006353487 |
| PI(34:0e)-H | PI | 1.843209273 | 0.004987958 |
| PE(16:1e_20:5)-H | PE | 1.839682723 | 0.023766485 |
| Cer(d33:0+O)+H | Cer | 1.836229621 | 0.028555848 |
| Cer(d22:0_20:0)+HCOO | Cer | 1.829012516 | 0.04040894 |
| PE(18:1_18:2)-H | PE | 1.826015379 | 0.02073404 |
| PE(18:1p_18:2)+H | PE | 1.818089724 | 0.000262058 |
| DG(34:2e)+H | DG | 1.813252916 | 0.021472385 |
| Cer(d44:1)+HCOO | Cer | 1.797997911 | 0.011831149 |
| PE(16:0p_22:4)+H | PE | 1.787590157 | 0.034110019 |
| PE(16:1_17:0)-H | PE | 1.783331774 | 0.042147282 |
| SM(d40:1)+HCOO | SM | 1.781377991 | 0.023750054 |
| GM3(d36:4)+HCOO | GM3 | 1.780075188 | 0.044914689 |
| PE(21:1)+H | PE | 1.771802175 | 0.006084252 |
| PC(20:0_16:0)+HCOO | PC | 1.768059595 | 0.016508657 |
| TG(18:0e_18:1_22:1)+NH4 | TG | 1.76692016 | 0.013700059 |
| PC(20:1e_10:0)+Na | PC | 1.760846515 | 0.038815239 |
| PC(17:1_16:0)+HCOO | PC | 1.759184605 | 0.023886611 |
| Hex1Cer(d18:1_16:0)-H | Hex1Cer | 1.740059223 | 0.031792218 |
| TG(16:0_12:0_14:0)+NH4 | TG | 1.734525467 | 0.043614321 |
| PC(15:0_16:1)+H | PC | 1.718655112 | 0.003225639 |
| Cer(d18:0_15:1)+HCOO | Cer | 1.7155514 | 0.000601725 |
| PS(18:3e_22:6)-H | PS | 1.709890786 | 0.006673531 |
| PS(18:1_22:3)-H | PS | 1.686592668 | 0.005994753 |
| PC(18:0_16:0)-CH3 | PC | 1.686398888 | 0.020857018 |
| PE(18:2e)+H | PE | 1.683025071 | 0.01153758 |
| PS(18:1_20:2)-H | PS | 1.68044245 | 0.013135137 |
| TG(18:1_18:1_18:1)+NH4 | TG | 1.659433979 | 0.046578937 |
| PS(40:5e)-H | PS | 1.656514146 | 0.033979249 |
| TG(16:0e_18:1_18:2)+NH4 | TG | 1.652235889 | 0.026031278 |
| PC(21:1_12:3)+H | PC | 1.645503433 | 0.035750252 |
| PS(34:3)-H | PS | 1.645403628 | 0.003848131 |
| LPC(30:1)+Na | LPC | 1.643897365 | 0.026494937 |
| DG(32:3e)+H | DG | 1.616268124 | 0.047568007 |
| TG(18:0_16:0_18:0)+NH4 | TG | 1.5923794 | 0.023070609 |
| TG(16:1_14:0_18:1)+NH4 | TG | 1.591941059 | 0.032020352 |
| PS(38:3e)-H | PS | 1.573389891 | 0.00707532 |
| TG(18:0_16:0_18:1)+NH4 | TG | 1.524840585 | 0.035813748 |
| PE(17:1)+H | PE | 1.519305773 | 0.017725004 |
| SM(d32:4)+H | SM | 1.502170841 | 0.014881325 |

**Table S3.** Significantly different lipid molecules analysed by multivariate statistical analysis.

| DOWN(EVs-EV71 vs EVs-Mock) |  |  |  |  |
| --- | --- | --- | --- | --- |
| **Lipid molecules** | **Class** | **Fold Change** | **P-value** | **VIP** |
| PC(30:0)+H | PC | 0.02249025 | 4.48124E-06 | 15.3382151 |
| Cer(m38:2)+NH4 | Cer | 0.292726919 | 0.000162341 | 1.179107314 |
| PE(40:7e)+Na | PE | 0.354045482 | 0.003071945 | 1.044040153 |
| PC(23:1_11:2)+H | PC | 0.498921397 | 0.002603845 | 2.609837711 |
| SM(d18:0_20:4)+H | SM | 0.511832757 | 0.038804843 | 1.001800824 |
|  |  |  |  |  |
| UP(EVs-EV71 vs EVs-Mock) |  |  |  |  |
| **Lipid molecules** | **Class** | **Fold Change** | **P-value** | **VIP** |
| SPH(d18:2)+Na | SPH | 37.53096778 | 0.034640971 | 1.435446456 |
| PG(18:1_14:0)-H | PG | 21.49413602 | 0.01812982 | 1.62833616 |
| LPC(20:4)+H | LPC | 19.59769458 | 0.003371336 | 1.071454781 |
| PE(38:3e)-H | PE | 14.21984597 | 0.002457011 | 3.165707483 |
| PE(16:0p_20:1)+H | PE | 11.72941751 | 0.043230517 | 7.533081504 |
| WE(2:0_16:2)+NH4 | WE | 10.16747375 | 0.00187772 | 1.084842747 |
| DG(38:5e)+H | DG | 9.891131019 | 0.000200108 | 1.017276085 |
| DG(12:0e_24:2)+Na | DG | 9.513713506 | 0.000373557 | 1.026039186 |
| LPC(18:1)+HCOO | LPC | 8.850670646 | 0.000170621 | 1.400166911 |
| PG(18:1_18:3)-H | PG | 8.173316466 | 7.60643E-07 | 1.025782761 |
| PE(16:0p_20:3)+H | PE | 8.146676461 | 4.81465E-08 | 1.985397358 |
| PI(18:0_20:3)-H | PI | 8.12470395 | 0.005929603 | 1.471324023 |
| PG(16:1_18:1)-H | PG | 7.839780804 | 0.015460707 | 2.008320417 |
| DG(18:0_20:3)+NH4 | DG | 7.113022283 | 0.014166218 | 1.117549771 |
| PC(32:4)+H | PC | 7.088687787 | 0.006160656 | 1.819627326 |
| TG(12:0e_6:0_22:6)+H | TG | 6.874530713 | 0.018849323 | 1.011816225 |
| PC(8:1e_10:0)+H | PC | 6.495963289 | 9.51837E-05 | 2.443218679 |
| LPC(16:0)+HCOO | LPC | 5.707636793 | 0.000134436 | 1.31606392 |
| PE(12:0p_23:1)+H | PE | 5.678760918 | 0.00062171 | 2.00229009 |
| LPE(18:1)-H | LPE | 5.621809989 | 0.000596034 | 1.520977505 |
| PE(41:2e)+H | PE | 5.451693202 | 0.000972607 | 2.255662988 |
| PE(16:1e_20:3)-H | PE | 5.445918348 | 0.001010389 | 1.569505505 |
| PE(12:0p_23:0)+H | PE | 5.347922896 | 0.006257209 | 6.752163443 |
| LPC(28:0)+H | LPC | 5.209418875 | 0.000351276 | 1.105937488 |
| SM(d34:4)+H | SM | 5.08004994 | 0.012687062 | 1.142324785 |
| PE(35:2e)+H | PE | 4.981545361 | 0.000577944 | 1.989735765 |
| PE(18:1p_22:2)+H | PE | 4.956632154 | 0.000558808 | 1.125605268 |
| PC(16:1_16:1)+HCOO | PC | 4.822342544 | 0.003673053 | 1.014824903 |
| PE(16:0p_22:3)+H | PE | 4.765682274 | 0.008273405 | 1.705633548 |
| PE(18:1e_20:3)-H | PE | 4.683227847 | 0.019876125 | 1.64061648 |
| PC(36:0e)+H | PC | 4.674184237 | 1.30148E-06 | 1.139126546 |
| PE(16:0p_22:2)+H | PE | 4.662895264 | 0.024749415 | 2.389509542 |
| PG(16:0_18:1)-H | PG | 4.557935042 | 0.030116049 | 1.168869283 |
| Cer(m19:0_23:3)+H | Cer | 4.548900797 | 1.26197E-06 | 1.118256657 |
| GM2(d34:1)-H | GM2 | 4.52917393 | 0.023204368 | 1.761919556 |
| Cer(t18:0_24:1)+H-H2O | Cer | 4.367275422 | 0.004401369 | 1.13315512 |
| PC(20:0_11:2)+H | PC | 4.271782745 | 1.78306E-06 | 2.705327392 |
| PE(18:2e_20:1)-H | PE | 4.158695746 | 0.025301015 | 2.291954049 |
| PG(18:1_18:2)-H | PG | 4.074249783 | 0.0158102 | 1.11629754 |
| PC(17:1_18:1)+H | PC | 4.066325349 | 1.10665E-06 | 1.367374846 |
| PE(16:0p_16:1)+H | PE | 4.062946301 | 4.37257E-06 | 2.736926544 |
| Cer(d18:1_24:1)+HCOO | Cer | 4.047294405 | 0.004010781 | 1.382134899 |
| PE(18:1p_22:1)+H | PE | 4.041754235 | 0.023882111 | 1.039644991 |
| LPE(18:1)-H | LPE | 4.025548569 | 8.38597E-06 | 1.144667172 |
| PS(17:0_18:1)-H | PS | 4.015454864 | 0.007005386 | 1.999956908 |
| PC(15:0_18:1)+H | PC | 4.003045293 | 4.90834E-07 | 2.284242768 |
| StE(24:7)+H | StE | 3.985322161 | 2.61537E-06 | 2.224205414 |
| Hex1Cer(m19:0_23:3)+H | Hex1Cer | 3.962569494 | 0.005505011 | 1.276998213 |
| PC(18:1_14:1)+HCOO | PC | 3.898997088 | 0.012358109 | 1.87017747 |
| PC(29:1)+H | PC | 3.887551753 | 1.02501E-05 | 1.487284562 |
| PE(18:2e_22:1)-H | PE | 3.864265958 | 0.006081741 | 1.324304169 |
| PG(18:1_18:1)-H | PG | 3.849653513 | 0.016416925 | 2.82466675 |
| DG(18:0_18:1)+NH4 | DG | 3.820960485 | 0.046929702 | 1.01303686 |
| PE(16:0p_22:1)+H | PE | 3.783589405 | 1.54336E-05 | 1.908547218 |
| PC(16:2e_19:0)+H | PC | 3.782342121 | 1.65779E-05 | 1.906535426 |
| PE(18:1p_20:4)+H | PE | 3.716321662 | 3.87552E-06 | 2.576753226 |
| CL(70:3)-2H | CL | 3.710166131 | 0.001988289 | 2.043050124 |
| Hex1Cer(d18:1_22:0)-H | Hex1Cer | 3.69498399 | 0.007144252 | 1.356332146 |
| PE(39:2e)+H | PE | 3.66289697 | 0.000976183 | 5.338016095 |
| PC(16:1_14:0)+HCOO | PC | 3.659130472 | 0.004709364 | 1.251172945 |
| LPE(16:1e)-H | LPE | 3.630765938 | 7.67748E-06 | 1.152485375 |
| PE(18:1p_22:3)+H | PE | 3.605749005 | 1.00062E-05 | 1.040711082 |
| GM3(d34:1)-H | GM3 | 3.57531068 | 0.016102466 | 2.22672876 |
| SPH(d17:0)+H | SPH | 3.569972172 | 9.54066E-05 | 3.251912884 |
| PE(18:1p_18:1)+H | PE | 3.52097609 | 0.006358241 | 3.301431992 |
| PE(16:1_18:1)-H | PE | 3.481712738 | 0.010160434 | 1.842365505 |
| PE(18:1_14:0)-H | PE | 3.473824321 | 0.009823951 | 1.067799536 |
| PE(18:1p_20:5)+H | PE | 3.449309771 | 3.74152E-06 | 1.474666019 |
| PE(12:0p_6:0)+H | PE | 3.430617886 | 0.001092769 | 1.03675582 |
| TG(48:12e)+Na | TG | 3.422338924 | 0.000558825 | 1.724994625 |
| PE(18:2e_18:1)-H | PE | 3.387929875 | 0.009459838 | 3.720895868 |
| PE(16:1e_22:1)-H | PE | 3.3830398 | 0.006725744 | 1.590902829 |
| PC(16:0e_16:1)+HCOO | PC | 3.355815128 | 0.002383605 | 1.334487146 |
| PE(16:0p_20:1)+H | PE | 3.348631325 | 3.23156E-05 | 3.541471331 |
| SPH(d19:0)+H | SPH | 3.335816944 | 8.15538E-07 | 2.546404578 |
| PC(33:2)+H | PC | 3.218227455 | 0.049184805 | 3.894569809 |
| PI(18:1_18:1)-H | PI | 3.204152453 | 0.04180627 | 1.223786124 |
| PC(33:2)+H | PC | 3.145279385 | 6.58229E-05 | 3.932687347 |
| PE(16:0p_18:1)+H | PE | 3.126206309 | 8.70459E-07 | 4.84018502 |
| PC(16:0e_20:1)+HCOO | PC | 3.099604815 | 0.008466489 | 1.002323514 |
| Cer(d18:1_16:0)+H | Cer | 3.087348818 | 0.002162577 | 1.306888809 |
| PE(18:0_18:1)-H | PE | 2.988864425 | 0.005931084 | 1.469346257 |
| Cer(d18:1_16:0)+HCOO | Cer | 2.965695745 | 0.000213378 | 1.303004114 |
| PI(18:0_18:1)-H | PI | 2.959525823 | 0.018067025 | 1.15059361 |
| Hex1Cer(d18:1_24:0)-H | Hex1Cer | 2.935912678 | 0.016181175 | 1.23753379 |
| Hex1Cer(d18:1_24:1)-H | Hex1Cer | 2.898188976 | 0.012363501 | 1.65356541 |
| PC(18:1_18:1)+HCOO | PC | 2.878901689 | 0.000230509 | 1.630938081 |
| PE(18:0e_18:1)-H | PE | 2.8727262 | 0.011059047 | 1.074031423 |
| PE(16:0p_20:5)+H | PE | 2.8431741 | 2.64315E-06 | 1.185244565 |
| PE(18:1_18:1)-H | PE | 2.695981283 | 0.014727008 | 2.166593305 |
| PE(16:1e_16:1)-H | PE | 2.693967241 | 0.003576641 | 1.313542931 |
| PC(16:0_20:1)+HCOO | PC | 2.681138958 | 0.027527143 | 1.26983861 |
| PE(37:1e)+H | PE | 2.662129765 | 0.002997451 | 5.791094343 |
| PC(16:1_18:1)+HCOO | PC | 2.655114687 | 0.013503685 | 2.113421164 |
| CL(70:1)-2H | CL | 2.652899618 | 0.001515328 | 1.263366553 |
| ZyE(24:7)+H | ZyE | 2.637124327 | 5.83051E-05 | 2.521635154 |
| PE(16:0_18:1)-H | PE | 2.597613516 | 0.000968791 | 1.161213696 |
| PC(16:0e_18:1)+HCOO | PC | 2.592087765 | 0.005656368 | 1.650443286 |
| PC(16:0_16:1)+HCOO | PC | 2.581179945 | 0.001385685 | 1.637227889 |
| PE(16:1e_20:1)-H | PE | 2.571630745 | 0.006898647 | 2.190972212 |
| CL(74:3)-H | CL | 2.569394188 | 0.007810842 | 1.888768095 |
| SM(d42:2)+HCOO | SM | 2.5011148 | 0.01006719 | 1.316011551 |
| PE(16:1e_20:4)-H | PE | 2.471417288 | 0.037085958 | 1.233125016 |
| PE(18:1e_20:4)-H | PE | 2.405041125 | 0.031744198 | 1.060967638 |
| PC(16:0_18:1)+HCOO | PC | 2.346388388 | 0.022931545 | 1.87344953 |
| phSM(t36:1)-CH3 | phSM | 2.30457322 | 0.006192244 | 1.498790981 |
| PE(16:1e_18:1)-H | PE | 2.244042479 | 0.009811661 | 1.895981924 |
| PC(16:0_16:0)+HCOO | PC | 2.068615894 | 0.032228656 | 1.482193772 |
| PC(16:0_14:0)+HCOO | PC | 2.014204293 | 0.026844126 | 1.214228043 |
| PE(18:1p_18:2)+H | PE | 1.818089724 | 0.000262058 | 1.13131812 |
| PE(16:0p_22:4)+H | PE | 1.787590157 | 0.034110019 | 1.025973012 |
| Hex1Cer(d18:1_16:0)-H | Hex1Cer | 1.740059223 | 0.031792218 | 2.270370015 |
